# Surface-Enriched Boron-Doped TiO_2_ Nanoparticles as Photocatalysts for Propene Oxidation

**DOI:** 10.1021/acsanm.2c02217

**Published:** 2022-08-24

**Authors:** L. Cano-Casanova, A. Ansón-Casaos, J. Hernández-Ferrer, A. M. Benito, W. K. Maser, N. Garro, M. A. Lillo-Ródenas, M. C. Román-Martínez

**Affiliations:** † Grupo Materiales Carbonosos y Medio Ambiente, Departamento de Química Inorgánica e Instituto Universitario de Materiales (IUMA), Facultad de Ciencias, Universidad de Alicante, Ap.99, E-03080 Alicante, Spain; ‡ Instituto de Carboquímica, ICB-CSIC, Miguel Luesma Castán 4, 50018 Zaragoza, Spain; § Institut de Ciència dels Materials (ICMUV), Universitat de València, 46980 Paterna, València, Spain

**Keywords:** photocatalysis, titanium dioxide, boron, interstitial doping, propene oxidation, photoelectrochemical characterization

## Abstract

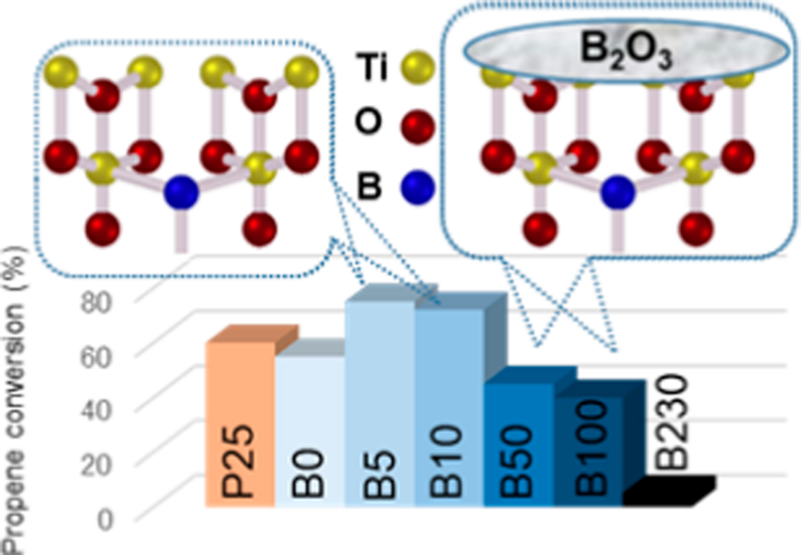

A series of nanostructured boron-TiO_2_ photocatalysts (B-*X*-TiO_2_-*T*) were prepared by sol–gel synthesis using titanium tetraisopropoxide and boric acid. The effects of the synthesis variables, boric acid amount (*X*) and crystallization temperature (*T*), on structural and electronic properties and on the photocatalytic performance for propene oxidation, are studied. This reaction accounts for the remediation of pollution caused by volatile organic compounds, and it is carried out at low concentrations, a case in which efficient removal techniques are difficult and costly to implement. The presence of boric acid during the TiO_2_ synthesis hinders the development of rutile without affecting the textural properties. X-ray photoelectron spectroscopy analysis reveals the interstitial incorporation of boron into the surface lattice of the TiO_2_ nanostructure, while segregation of B_2_O_3_ occurs in samples with high boron loading, also confirmed by X-ray diffraction. The best-performing photocatalysts are those with the lowest boron loading. Their high activity, outperforming the equivalent sample without boron, can be attributed to a high anatase and surface hydroxyl group content and efficient photo-charge separation (photoelectrochemical characterization, PEC), which can explain the suppression of visible photoluminescence (PL). Crystallization at 450 °C renders the most active sample, likely due to the development of a pure anatase structure with a large surface boron enrichment. A shift in the wavelength-dependent activity profile (PEC data) and the lowest electron–hole recombination rate (PL data) are also observed for this sample.

## 1 Introduction

Environmental legislation drives increasing emphasis on removing toxic organic pollutants, like volatile organic compounds (VOCs), from air.^1^ Among VOCs, propene is one of the main components of tobacco smoke, and it is present in vehicle emissions and exhaust gases from several industries (petrochemicals, foundry processes, etc.).^2,3^ It has important harmful effects on human health, even at low concentrations. Photocatalysis has shown to be one of the most suitable techniques to successfully degrade organic pollutants both in the gas and the liquid phase (leading to its complete mineralization).^4−6^

Titanium dioxide (TiO_2_) is considered an excellent and widely used photocatalyst, with outstanding properties like high stability, resistance to corrosive media, good biocompatibility, and relatively low cost due to the abundance of titanium on the earth crust.^7−11^ These properties and issues confer to TiO_2_ advantages over other semiconductors like ZnO, CeO_2_, CdS, ZnS, and so forth.^12,13^ However, its photocatalytic efficiency is hindered by the fast recombination of photogenerated holes and electrons and its relatively wide band gap (3.2 eV) that limits its activity to the near-UV region^14^ and, thus, only about 5% of solar irradiation can be used.^15,16^ Many research studies have focused on combining TiO_2_ with other elements or compounds with the purpose of increasing the TiO_2_ efficiency.^17,18^

Recently, boron incorporation into nanostructured TiO_2_ has been proposed as an alternative to overcome some of the drawbacks mentioned above. A potential benefit of the presence of boron is the creation of electronic levels that could hinder the recombination of the electron–hole pairs.^16,18−26^ Density functional theory calculations suggest that the occupation of interstitial sites or O substitution are more energetically favorable than substitution of Ti by B atoms.^24^ It has been reported that the addition of boron to TiO_2_ sol–gel precursors generally leads to interstitial B-doping, together with the formation of B_2_O_3_ on the surface of the TiO_2_ particles.^16,19,27,28^ Some studies have also shown that surface boron species can introduce residual charge, which can increase the number of surface OH groups, being them correlated with the incorporated B.^19,29^

The positive influence of boron has also been related to changes in the TiO_2_ structure as it could inhibit the growth of crystalline TiO_2_, leading to materials with increased surface area^30^ and anatase content.^18^

Several studies have reported a photoactivity improvement upon B doping of TiO_2_,^16,18−26^ but the interpretation is still controversial, especially regarding the local structure around the boron impurity and the electronic effects of the dopant species.^16,19,31^ Zhang et al. reported highly efficient photocatalysts consisting of B-TiO_2_ (3.5 at. B %) for gas phase degradation of benzene under UV–vis light,^22^ which are more active than the commercial TiO_2_-P25. This improvement is ascribed to a significant enhancement of the UV–vis light absorption.^22^ Ansón-Casaos et al. prepared B-TiO_2_ photocatalysts by sol–gel synthesis using boric acid,^16^ leading to a slightly improved photocatalytic efficiency in the degradation of diphenhydramine (drug pollutant model) in water. UV–vis diffuse reflectance analysis allowed to discard visible light absorption, and the activity improvement was attributed to the crystalline structure and composition of the photocatalysts. Jeong et al.^19^ prepared a boron-doped TiO_2_ anode (sol–gel, 2, 5, and 10 wt % B) for lithium-ion batteries, and they found a significant improvement with respect to pristine TiO_2_, which was attributed to the increased interplanar spacing of the TiO_2_ lattice due to interstitial B atoms and/or to a larger amount of surface hydroxyl groups. Dozzi et al. studied B- and F- doped TiO_2_ materials, and they found that the photodegradation of formic and acetic acid (in the liquid phase) can be correlated with the photocatalyst’s structural features. The higher activity of the doped TiO_2_ with respect to the bare one was attributed to its higher surface area (from 45 to 100 m^2^/g) and the hindered transformation of anatase into rutile, whereas the surface B_2_O_3_ formed (observed by XPS) had no effect on the photoactivity.^27^

Many of the indicated effects can be positive, enhancing the photocatalytic activity of TiO_2_ for propene oxidation, which is the target reaction of the present study.

With the aim of shedding light on the role of boron in the physicochemical properties of TiO_2_ modified with this element, the present research focuses on the study of nanostructured B-TiO_2_ samples, prepared by sol–gel synthesis using titanium tetraisopropoxide and different amounts of boric acid and crystallization temperatures. A detailed characterization has been carried out using a plethora of techniques [gas adsorption, X-ray diffraction (XRD), X-ray photoelectron spectroscopy (XPS), UV–vis spectroscopy, analysis of photoluminescence (PL), photoelectrochemical characterization (PEC), and Raman scattering]. The prepared photocatalysts have been tested in the gas phase photocatalytic oxidation of propene (100 ppmv), which accounts for remediation of pollution caused by low-concentration VOCs, a case in which efficient removal techniques are difficult and costly to implement.^4,32^

The general mechanism of propene oxidation involves the following steps (where R represents the organic substrate)
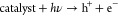
1
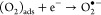
2
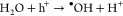
3
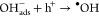
4
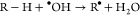
5
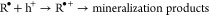
6

Reactions 5 and 6 are an example of the possible interaction of the substrate with oxidant species. In a general way: R–H + ^•^OH/O_2_^•–^/h^+^ → mineralization products.

In the case of propene oxidation, the global reaction can be written as
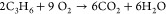
7

The thorough characterization of the B-TiO_2_ samples, using techniques that allow an approach to the photocatalyst properties from very different points of view and that will help to find a suitable correlation between properties and activity of the photocatalysts, is one of the main novelties of this study.

## 2 Experimental Section

### 2.1 Materials

Titanium(IV) isopropoxide (TTIP, C_12_H_28_O_4_Ti, 97%, Sigma-Aldrich), boric acid (H_3_BO_3_, 99.9%, Gilca), and 2-propanol (C_3_H_8_O, >99.8%, Panreac AppliChem) were used as-received. The well-known standard Aeroxide P25 TiO_2_ photocatalyst from Degussa was used as a reference.

### 2.2 Synthesis of B-TiO_2_ Photocatalysts

**Scheme 1:**
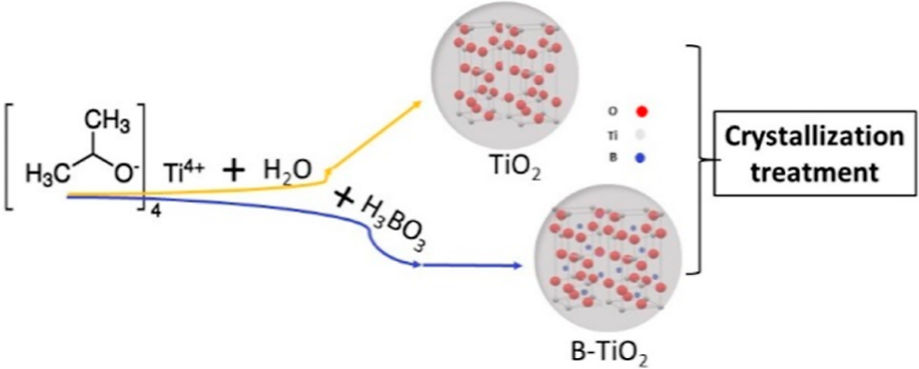
Illustration of the Photocatalysts Preparation and Composition/Structure

The synthesis procedure is based on a previously optimized sol–gel method.^16^ In a typical preparation, 5 mL of TTIP were diluted in 50 mL of isopropanol and added dropwise, under constant stirring, to 50 mL of a water/isopropanol (1:1) mixture containing a certain quantity of boric acid. The resulting gel was aged overnight, filtered, and dried in an oven at 90 °C. The as-prepared amorphous TiO_2_ was thermally treated in a horizontal quartz reactor under an ambient atmosphere to induce crystallization. The obtained materials are labeled as B-*X*-TiO_2_-*T*, where *X* refers to the amount of boric acid (mg) added during the synthesis and *T* (°C) to the crystallization temperature. Scheme 1 illustrates the preparation and structure of the catalysts.

The nominal B content in the resulting samples was estimated taking into account the yield of the TiO_2_ synthesis and assuming that all the boron from the added boric acid remains in the final product. More specifically, the average hydrolysis yield for 5 mL TTIP was 1.5 ± 0.1 g, and the yield of the thermal crystallization treatment was 81.5 ± 1.5%. Therefore, the boron content ranges from 0.07 to 3.4 wt %.

### 2.3 Characterization

N_2_ adsorption–desorption at −196 °C (Quantachrome Autosorb-6B Instrument) after sample degasification (250 °C, 4 h) was used to characterize the porous texture [the apparent Brunauer–Emmett–Teller (BET) specific surface area (*S*_BET_) and the total micropore volume (*V*_DR_ N_2_, ϕ < 2 nm), applying BET and Dubinin Radushkevich equations, respectively].^33^ An estimation of the mesopore volume (2 nm < ϕ < 50 nm, *V*_meso_) was calculated as *V*_N_2__ ads (*P*/*P*_0_ = 0.9) – *V*_N_2__ ads (*P*/*P*_0_ = 0.2), expressed as liquid.^34^ Total pore volume (*V*_T_) was determined as the volume of nitrogen adsorbed at *P*/*P*_0_ = 0.99 (as liquid).

XRD measurements were carried out in a Miniflex II Rigaku (30 kV/15 mA) equipment at room temperature, with Cu Kα radiation, 2°/min scanning rate, and 6–80° 2θ range. The average crystallite size (*B*) was calculated with the Scherrer equation^35^
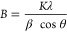
8where λ is 0.1540 nm (Cu Kα radiation wavelength), *K* is a constant (taken as 0.93 considering spherical grains),^36^ and β and θ are, respectively, the full width at half-maximum intensity (FWHM) and the diffraction angle of the main peak for each crystalline phase.

Crystalline TiO_2_ was characterized and quantified using XRD data of 50/50 (wt/wt) B-TiO_2_/CaF_2_ mixtures, as described in ref (37). Further explanations on the calculation of total crystallinity and percentage of crystalline phases are reported in Section 3.1.

High-resolution transmission electron microscopy (HRTEM) in combination with an energy-dispersive X-ray spectroscopy EDX (FEI Tecnai G2 system with a Schottky field emission electron gun operated at 200 kV) were used for characterizing TiO_2_ microstructure and chemical composition in some prepared samples. For that purpose, TiO_2_ nanopowders were suspended in ethanol and dispersed on TEM grids.

XPS was measured in a VG-Microtech Multilab 3000 spectrometer provided with an Al anode working at 6 mA and 12 kV and with a pass energy of 50 eV. Binding energies were referenced to the C 1s line at 284.6 eV.^38^

PL and Raman scattering spectra of cold-pressed pellets of the powder catalysts were recorded using a laser diode as the excitation source (λ_exc_ = 405 nm) and a ×50 microscope objective to achieve an irradiance of about 10^8^ W/m^2^. The laser light was rejected by a longpass edge filter, which cutoff all signals below 200 cm^–1^. Spectra were recorded with a HORIBA Jobin Yvon iHR320 spectrometer equipped with a Peltier-cooled CCD detector. All measurements were carried out at room temperature and atmospheric pressure with the samples in contact with air.

Diffuse reflectance measurements were carried out on a Shimadzu UV–vis 2501PC spectrophotometer equipped with an integrating sphere. BaSO_4_ was used as reference material and for background measurement. The measurement range studied was 220–900 nm, with a 1.0 nm resolution and a spectral bandwidth of 5 nm. The spectra recorded were transformed using the Kubelka–Munk function.

For the PEC characterization, powder photocatalysts were suspended in isopropanol using an ultrasound bath and immediately deposited on fluorinated tin oxide (Solems SA, 80 Ω/sq) substrates by spray coating.^39^ The as-prepared films were sintered at 450 °C for 2 h under ambient air. Specimens were connected as working electrodes in a three-electrode configuration, with a graphite bar as the counter electrode, the Ag/AgCl, 3 M NaCl (*E*° = 0.210 V vs SHE) as the reference electrode, and 0.1 M Na_2_SO_4_ as the electrolyte. The electrolyte was deoxygenated by flowing N_2_ for longer than 10 min before the measurements. The glass PEC cell was provided with a quartz window and irradiated with a laboratory-scale solar simulator (150 W Xe arc lamp by LOT-Quantum Design GmbH). The simulated solar spectrum was the AM1.5G with a maximum irradiance of approximately 100 mW/cm^2^ (see Figure S1A). The wavelength-dependent response was measured using a LOT Quantum Design monochromator (MSH-300).

### 2.4 Photocatalytic Oxidation of Propene

The photocatalytic tests of propene oxidation were carried out in an experimental setup that consists of a quartz reactor (AFORA) and a UV-A lamp (Philips, TL8 W/05 FAM, λ_max_ = 365 nm) located parallel to the photoreactor at a distance of about 1 cm. Some experiments have been carried out using a visible lamp (Sylvania, F8W/T5/54-765, Daylight). The spectra of both lamps can be seen in Figure S1, Supporting Information. A scheme of the setup is shown elsewhere,^2^ and a detailed description of the experimental conditions has been previously reported by Cano-Casanova et al.^40,41^ The UV-A irradiance of the Philips lamp was determined at 1 cm distance with a portable Delta Ohm photo radiometer (model HD 2102.2) equipped with a UV-A probe (LP 471 UVA), being 2.24 mW/cm^2^.

In a typical experiment, the photocatalyst (0.11 g) was placed on a quartz wool plug inside the reactor and, after purging with helium (60 mL/min), a stream of propene in air [100 ppmv propene, 30 or 60 mL/min (STP)] was passed through the reactor at 25 °C. The outlet stream was continuously monitored by mass spectrometry (Thermostar GSD 301 O1, from Balzers), and once the signal was stabilized, the UV-lamp was switched on, and the illumination was maintained for 3 h. The experiments were repeated at least twice to check reproducibility.

Blank experiments (in the absence of the catalyst) and measurements in dark show that propene conversion does not occur in these conditions.

**Scheme 2:**
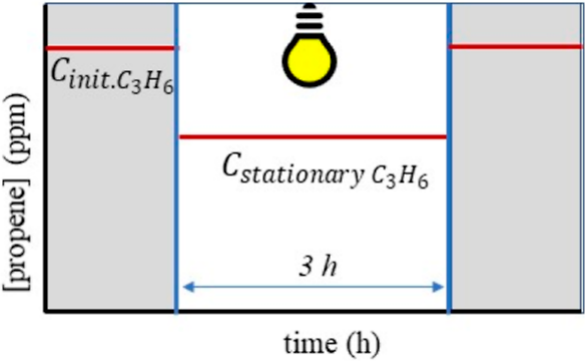
Drawing of the Variation of the Propene Concentration under Illumination (in Presence of a Photocatalyst), Parameters as Indicated Above.

Propene conversion was calculated, as shown in eq 9
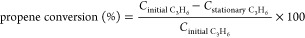
9where *C*_initial C_3_H_6__ is the initial propene concentration (100 ppmv) and *C*_stationary C_3_H_6__ is the stationary propene concentration under illumination (3 h reaction time). This is illustrated in Scheme 2.

Reaction products, i.e., carbon dioxide and water, were also analyzed by mass spectrometry. A calibration cylinder containing 300 ppmv CO_2_ in He was used for CO_2_ quantification. Mass scan measurements (up to *m*/*z* = 100) allowed to discard any additional oxidation compound, and the total mineralization of propene was confirmed by the carbon balance,^2,3^ according to the following reaction
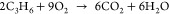


This condition was verified for each experiment.

## 3 Results and Discussion

This section includes the results of different characterization techniques with the aim of analyzing: (i) the effect of the amount of boric acid used and (ii) the effect of the crystallization temperature, on the photocatalytic activity.

### 3.1 Characterization of the Prepared Samples

#### 3.1.1 XRD Analysis and Textural Properties

**Figure 1:**
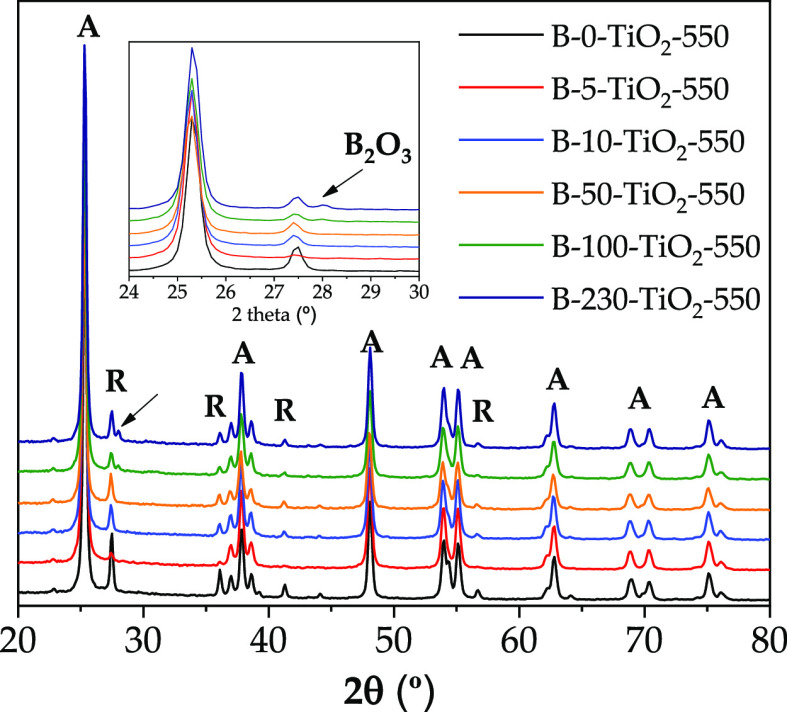
XRD spectra for B-*X*-TiO_2_-550 samples. Inset image: amplification of Figure 1 in the 24–30° 2θ range.

XRD patterns of the B-*X*-TiO_2_-550 samples (Figure 1) show the characteristic peaks of anatase [2θ values of 25.3° (101), 37.8° (004), 48.0° (200), 54.5° (105), 55° (211), 62.7° (204), 70.4° (116), and 74.5° (220)] and rutile [2θ values of 27.5° (110), 36.1° (101), and 54.4° (211)].^42,43^ B-100-TiO_2_-550 and B-230-TiO_2_-550 samples show an additional peak at 2θ = 28.1°, which corresponds to boric oxide (B_2_O_3_)^16,44^ (inset of Figure 1), resulting from the thermal decomposition of boric acid (boric acid decomposes at about 150 °C into boric oxide and water^45^). B_2_O_3_ might also be present in the other samples but it is not detected, either because the amount is too low or it has low crystallinity.

The XRD profiles of the B-*X*-TiO_2_-550 series (*X* ≠ 0) show that boron incorporation does not take place in the bulk TiO_2_ crystallite and, thus, it can be considered that boron atoms are not extensively inserted in the anatase or rutile unit cells.

The calculated percentage of crystalline and amorphous phases and the average crystallite size for all the prepared photocatalysts and P25 are listed in Table 1. The percentages of anatase and rutile have been calculated using the following equations
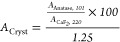
10
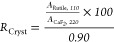
11where *A*_Anatase,101_, *A*_Rutile,110_, and *A*_CaF_2_,220_ are the areas determined from the XRD pattern.

**Table 1:** Crystalline Properties and Surface Area in the B-*X*-TiO_2_-550 Series and P25^a^

	crystalline TiO_2_			average crystallite size (nm)		
sample	A (%)	R (%)	amorphous TiO_2_ (%)	A	R	*S*_BET_ (m^2^/g)
B-0-TiO_2_-550	65	12	23	28	32	28
B-5-TiO_2_-550	75	2	23	25	19	33
B-10-TiO_2_-550	70	6	24	25	27	30
B-50-TiO_2_-550	56	6	38	25	29	24
B-100-TiO_2_-550	66	5	29	24	23	27
B-230-TiO_2_-550	70	7	23	27	25	25
P25	73	14	13	22	28	55

a A = anatase, R = rutile.

Thus, the percentages of crystalline and amorphous TiO_2_ are, respectively, *W*_Cryst_ = *A*_Cryst_ + *R*_Cryst_ and *W*_Am_ = 100 – *W*_Cryst_.

These data show that, in general, the presence of boric acid during the TiO_2_ synthesis hinders the formation of rutile, stabilizing up to some extent the anatase metastable phase, in agreement with the literature.^18,30^ Although differences between the B-*X*-TiO_2_-550 samples regarding phase composition are small, it can be highlighted that B-5-TiO_2_-550 is the one with the highest anatase and lowest rutile content, whereas B-50-TiO_2_-550 has the lowest anatase content and the highest proportion of amorphous TiO_2_.

The specific surface area (*S*_BET_) of the B-*X*-TiO_2_-550 samples is about half that of P25, ranging from 24 to 33 m^2^/g (see complete textural properties in Table S1, Supporting Information), and it does not significantly vary with the amount of boron present in the sample.

#### 3.1.2 HRTEM–EDX Characterization

The comparative TEM–HRTEM analysis of samples B-0-TiO_2_-550 and B-5-TiO_2_-550 (see Supporting Information, Figures S2 and S3) shows that the TiO_2_ nanoparticles have a similar particle size (50 nm average size), but some interesting differences in the particles structure have been observed. Thus, in sample B-0-TiO_2_-550 both monocrystal and some polycrystalline nanoparticles are present, and most of them also show amorphous domains, whereas B-5-TiO_2_-550 contains mainly monocrystals.

EDX analysis (Figure S2) has shown no differences between B-5-TiO_2_-550 and B-0-TiO_2_-550 samples. The lack of detectable signal at the K-alpha emission of boron (expected at 0.183 keV) in the spectrum of B-5-TiO_2_-550 in not surprising due to the low boron content in this sample and the proximity of the K-alpha peak of C (carbon is present in the TEM grids and produces a strong peak).

#### 3.1.3 XPS Analysis

**Figure 2:**
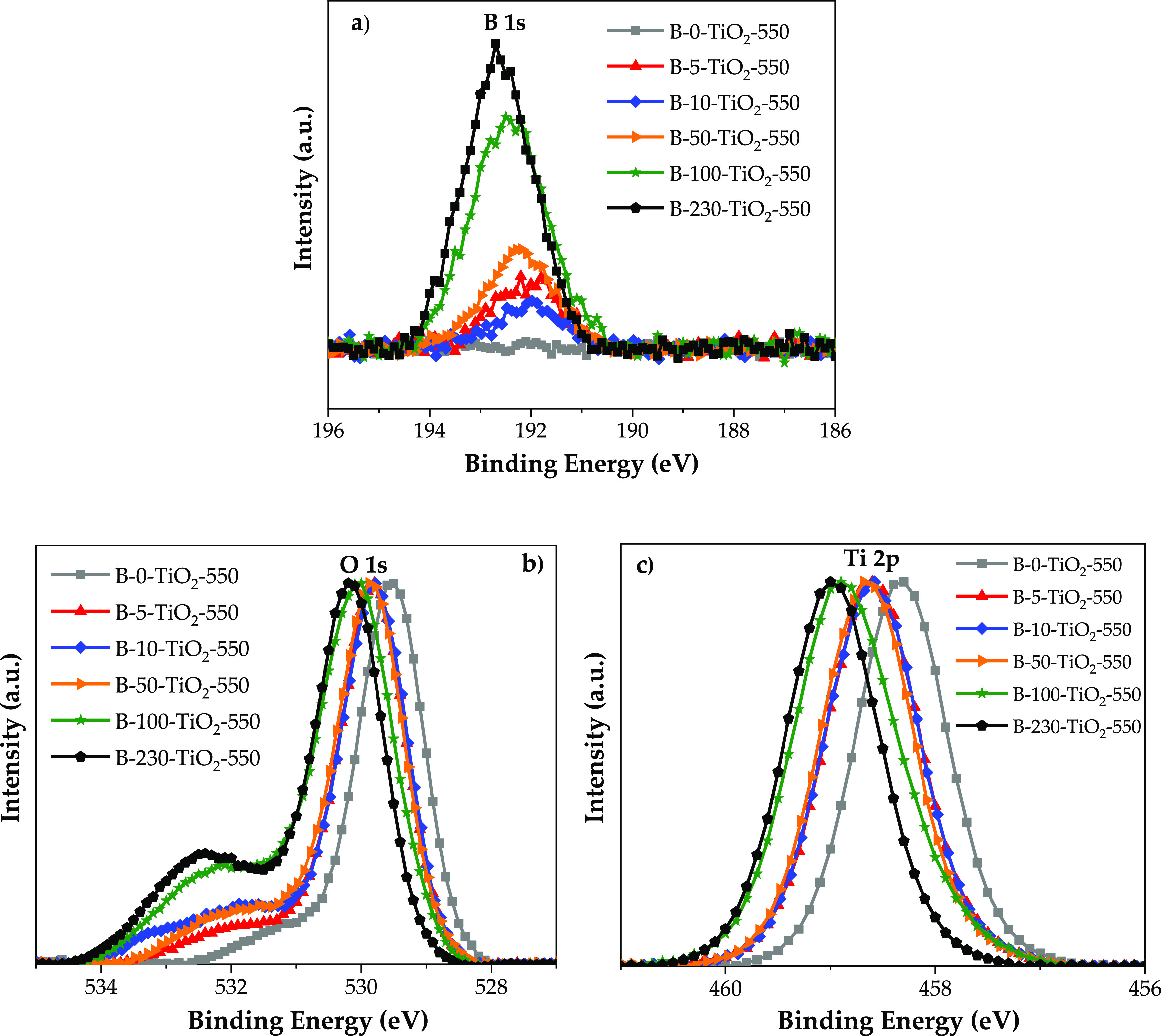
XPS data of (a) B 1s, (b) O 1s, and (c) Ti 2p for the B-*X*-TiO_2_-550 samples. The original data are plotted for B 1s, whereas for O 1s and Ti 2p, normalized data are presented (to better observe the shifts).

The XPS spectra survey of the B-*X*-TiO_2_-550 samples series is presented in Figure S4. Figure 2a–c shows the XPS spectra of the B-*X*-TiO_2_-550 photocatalysts for B 1s, O 1s, and Ti 2p, respectively (the latter in the 461–456 eV range, see full-range Ti 2p spectra in Figure S5). The corresponding binding energy (BE) values are collected in Table 2. Note that these values correspond to the maxima of the experimental spectra except for O 1s, for which three binding energies have been obtained from the fitted deconvoluted spectra of each oxygen contribution.

**Table 2:** Binding Energies of B 1s, O 1s, and Ti 2p for B-*X*-TiO_2_-550 Samples

	BE (eV)						
sample	B 1s	O 1s (I)	O 1s (II)	O 1s (III)	Ti 2p_3/2_	B at%	B at. % (nom)^a^
B-0-TiO_2_-550		529.6	531.1		458.3		0.00
B-5-TiO_2_-550	192.1	529.8	531.2	532.4	458.8	2.8	0.14
B-10-TiO_2_-550	192.1	529.8	531.5	532.8	458.7	2.3	0.29
B-50-TiO_2_-550	192.2	529.8	531.3	532.4	458.7	6.0	1.41
B-100-TiO_2_-550	192.8	530.0	531.5	532.6	459.0	8.9	2.78
B-230-TiO_2_-550	192.8	530.2	532.3	532.9	459.0	10.2	6.12

a Nominal atomic percentage of boron, calculated according to the boric acid used and the yield of the preparation method.

As shown in Figure 2a and in Table 2, the B 1s BE is around 192.1 eV in B-5-TiO_2_-550, B-10-TiO_2_-550, and B-50-TiO_2_-550 samples, while it is 192.8 eV in B-100-TiO_2_-550 and B-230-TiO_2_-550. As the B 1s BE in B_2_O_3_ is 192.9 eV,^46,47^ it can be assumed that boron is largely present as B_2_O_3_ on the surface of the B-100-TiO_2_-550 and B-230-TiO_2_-550 samples, which is in agreement with the presence of B_2_O_3_ determined by XRD (see Figure 1). In the case of the B-5-TiO_2_-550, B-10-TiO_2_-550, and B-50-TiO_2_-550 samples, boron is present in a less deficient electronic state, which can be related to a lower interaction with electronegative atoms such as O. Quesada-González et al.^48^ in their study of B-TiO_2_ samples reported that the B 1s BE of about 192 eV corresponds to interstitial B and that a peak at 192.6 eV can be attributed to H_3_BO_3_ or B_2_O_3_. The assignation of a 191.8 eV BE to interstitial B is also reported by Feng et al.^21^ and Zhang et al.,^22^ who also found a 191.9 eV BE for B-TiO_2_ samples. They proposed that B is incorporated into the TiO_2_ lattice and that its chemical environment might be Ti–O–B.^22^

These results evidence the presence of interstitial B in our photocatalysts, being easily detected by XPS analysis on the surface of the samples with lower B loading, whereas it could probably be covered by B_2_O_3_ in the B-100-TiO_2_-550 and B-230-TiO_2_-550 samples. In the study of Quesada-González et al.,^48^ the interstitial boron was detected by in-depth XPS analysis.

The O 1s spectra show that oxygen is present in different electronic states. In the case of B-0-TiO_2_-550, the main peak is due to O bonded to Ti (at 529.6 eV, TiO_2_ lattice oxygen), and a shoulder (at 531.1 eV) corresponding to O in surface OH groups^22^ can be observed [identified as O(I) and O(II), respectively] in Table 2.

**Figure 3:**
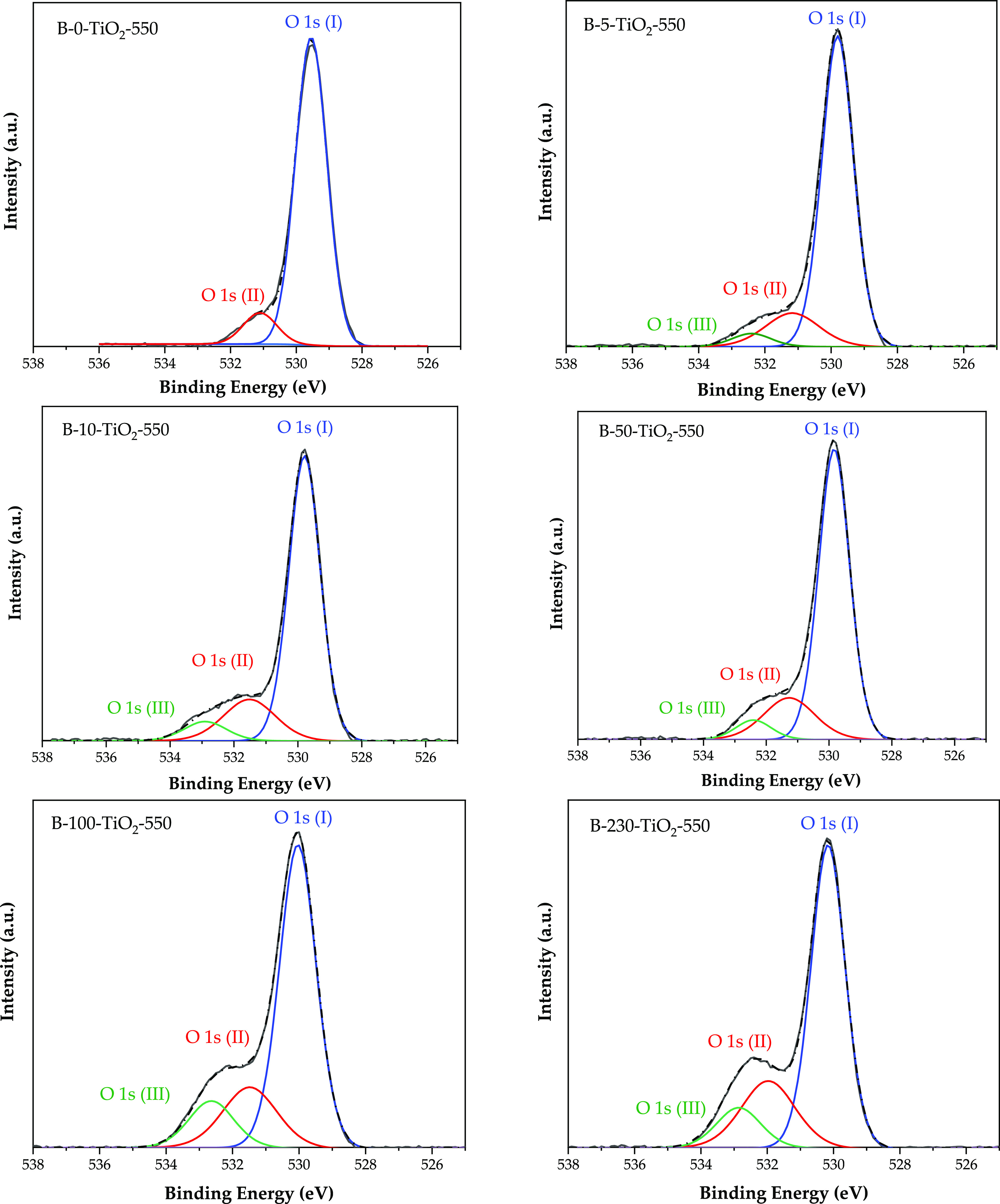
O 1s XPS deconvoluted spectra for the B-*X*-TiO_2_-550 samples.

For the boron-containing samples, apart from the main O–Ti peak, a two-component band, with its maximum at BE ≥ 532 eV, can be observed. It corresponds to either O bonded to boron^22^ or O in OH surface groups.^22^ Such a band has been deconvoluted to distinguish these two contributions (Figure 3), and the one assigned to O–B has been identified as O(III) in Table 2. As in the case of the B1s data, there are differences between B-100-TiO_2_-550 and B-230-TiO_2_-550 samples on one side, and B-5-TiO_2_-550, B-10-TiO_2_-550, and B-50-TiO_2_-550 on the other side, both in the intensity and in the BE of these O–B species. In agreement with the higher B content, the proportion of O bonded to B species (O(III)) is noticeably higher in the B-100-TiO_2_-550 and B-230-TiO_2_-550 samples and, in line with B 1s BE and XRD data, these species largely correspond to B_2_O_3_ on the TiO_2_ surface. In the case of the B-5-TiO_2_-550, B-10-TiO_2_-550, and B-50-TiO_2_-550 samples, the O–B bonds should correspond to the surface interstitial species present in these samples.

The BE of Ti 2p_3/2_ in sample B-0-TiO_2_-550 (458 eV) shows that, as expected, titanium is present as Ti^4+^ (Figures 2 and S5). For the B-*X*-TiO_2_-550 series of samples, the BE is slightly shifted to higher values, indicating that the presence of boron reduces the electron density of Ti 2p.^26^ This can be a consequence of the proximity of boron atoms (more electronegative than Ti) located in interstitial positions of the TiO_2_ structure. The displacement seems to increase with the boron amount, being related to more boron atoms located in interstices. It is important to point out that the distortion of the TiO_2_ lattice produced as a consequence of boron insertion has been detected by means of XPS data and not by XRD, which indicates that it is not a bulk extensive phenomenon. Then, it could be assumed that it happens mainly in a surface region. The B_2_O_3_ identification by XRD analysis allows considering that only a certain amount of boron can be hosted in the TiO_2_ lattice, and the rest is deposited on the surface of the TiO_2_ nanoparticles as B_2_O_3_.

### 3.2 Photocatalytic Activity

**Figure 4:**
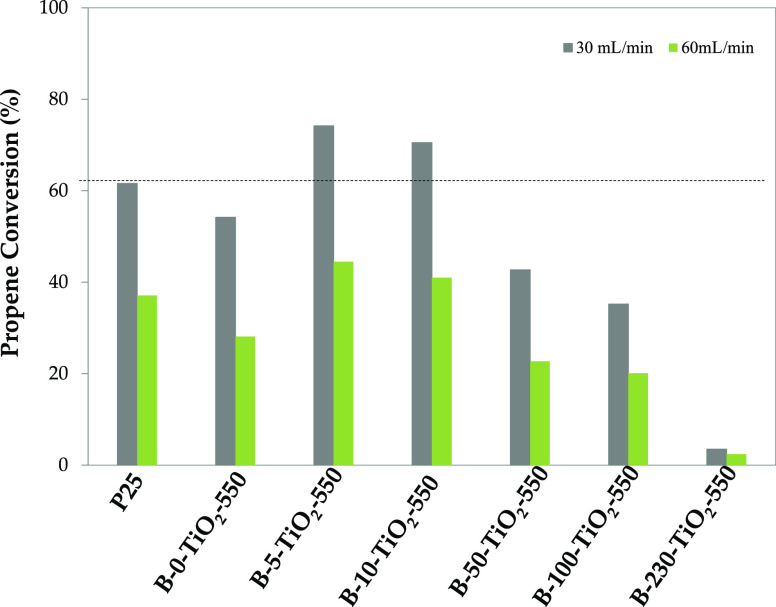
Propene conversion (%) at 30 and 60 mL/min for B-*X*-TiO_2_-550 photocatalysts and P25.

Figure 4 shows propene conversion data obtained with the two flow rates tested. Photolysis does not occur, and these results indicate that all the prepared photocatalysts are active, being two of them more active than the reference P25. Furthermore, the same trend is found with the two propene flow rates, which proves the reproducible performance of these catalysts. The lower flow rate leads to a higher conversion because of the larger contact time and/or the lower amount of propene molecules to be oxidized per unit of time.

The catalyst stability has been stated after confirming that the MS propene signal remains stable during the whole experiment. As no decay was observed at all, it can be assumed that the catalysts remain stable.

The order in the activity of B-*X*-TiO_2_-550 samples is: B-5-TiO_2_-550 > B-10-TiO_2_-550 > B-0-TiO_2_-550 > B-50-TiO_2_-550 > B-100-TiO_2_-550 > B-230-TiO_2_-550.

Samples B-5-TiO_2_-550 and B-10-TiO_2_-550 are more active than the prepared bare TiO_2_ (sample B-0-TiO_2_-550) and also than P25. However, B-*X*-TiO_2_-550, with *X* = 50, 100, and 230 samples are less active, and the photocatalytic activity decreases as the amount of boric acid used increases, being sample B-230-TiO_2_-550 practically inactive. These results imply that interstitial boron doping likely leads to electronic modifications that improve the TiO_2_ photoactivity. However, above a certain amount of boron, there is an excess that segregates as B_2_O_3_ and partially covers the interstitially boron-doped TiO_2_ surface, with the consequent photocatalytic activity decrease. Thus, the very good photocatalytic performance, shown by B-5-TiO_2_-550 and B-10-TiO_2_-550, seems to be related to the low, but suitable, quantity of boron present and its location. The B-5-TiO_2_-550 sample contains the highest anatase and the lowest rutile amount, and it has a certain content of surface interstitial boron. These characteristics likely enhance the photocatalytic propene oxidation.

Some selected samples were also tested under visible light. The results are discussed in Section 3.3, but it can be anticipated that the activity is significantly lower. Propene conversion (using 30 mL/min propene flow) of sample B-5-TiO_2_-550 decreases from 74% (under UV light) to 18% (under visible light) (compare data in Figures 4 and S10).

In order to better understand and to explain why the photocatalysts that contain the lowest amount of boron show the best photocatalytic activity, they were further characterized by means of PEC and PL measurements.

#### 3.2.1 PEC and PL Characterization of the Most Active Samples

**Figure 5:**
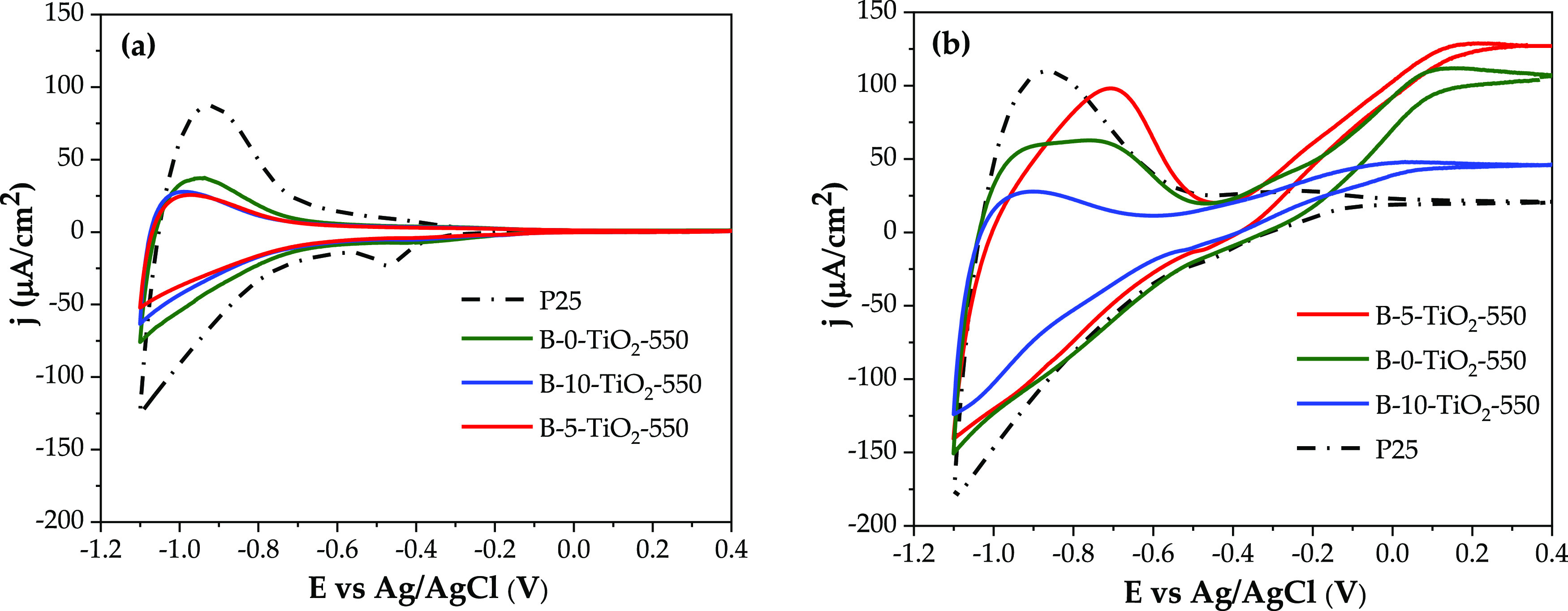
CV scans in the dark (a) and under full solar irradiation (b) using various TiO_2_ specimens as working electrodes. Scan rate = 20 mV s^–1^, irradiation intensity ∼100 mW cm^–2^.

Figure 5 shows the analysis by cyclic voltammetry (CV) in the dark and under irradiation in a solar simulator. In the dark, the shape of the CV scans reflects the accessibility of electronic energy states that are near the conduction band level,^49^ which is located at around −1 V versus Ag/AgCl. The CV plot of P25 is quite different from that of the B-*X*-TiO_2_-550 samples tested. Specifically, the hysteresis of P25 at potentials below −0.7 V versus Ag/AgCl is wider, which can be associated to the higher *S*_BET_ and, thus, to a larger charge accumulation on the surface. Besides, trap states around −0.5 V are more clearly defined for P25, in particular in the backward (negative currents) scan. All the B-*X*-TiO_2_ samples tested show a similar scan shape, as all of them have close *S*_BET_ values and, thus, similar charge accumulation capacities.

Under full solar spectrum irradiation, a photocurrent is observed at *E* > −0.35 V for the tested B-*X*-TiO_2_-550 samples, being the highest one for B-5-TiO_2_-550 and B-0-TiO_2_-550 anodes. Besides, both electrodes show a particular distortion in the charge accumulation region, with a clear feature at around −0.7 V (positive currents scan). This feature points to the creation under irradiation of certain surface states close to the conduction band, being particularly pronounced for B-5-TiO_2_-550, which could explain the better photocatalytic performance of this sample.

**Figure 6:**
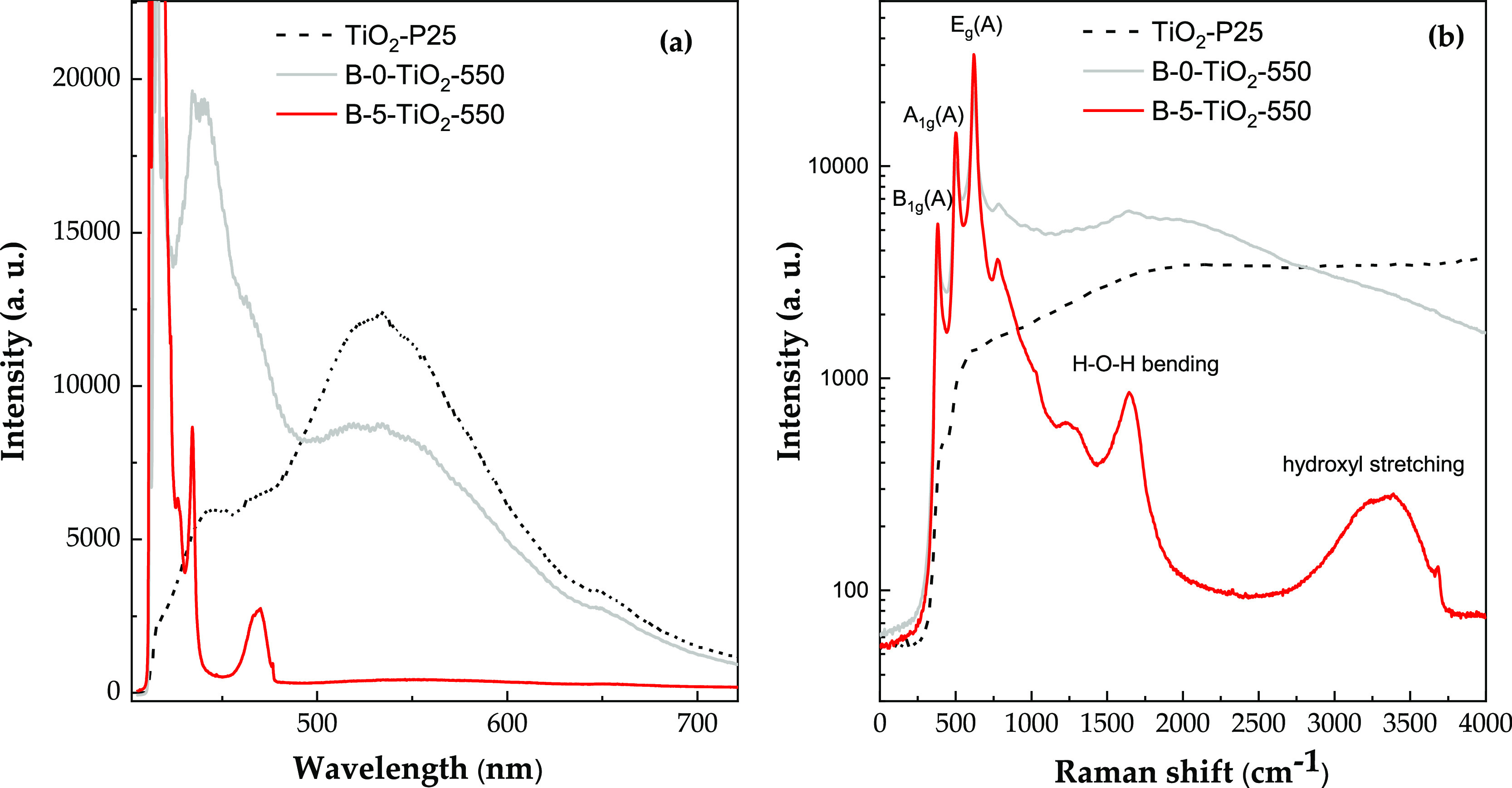
(a) PL spectra of B-0-TiO_2_-550, B-5-TiO_2_-550, and TiO_2_-P25. (b) Expansion of the spectral range between 405 and 485 nm, showing the Raman scattering spectra in detail (the scale has been changed to wavenumbers, and intensity is represented in the logarithmic scale, for clarity).

Figure 6a shows the PL spectra obtained for B-0-TiO_2_-550, B-5-TiO_2_-550, and P25, with an excitation source of 405 nm (see Figure S6 for the PL spectra of the complete B-*X*-TiO_2_-550 series). This excitation energy is slightly lower than rutile or anatase TiO_2_ band-gap energies and, therefore, the absorption should correspond to transitions from the valence band to shallow trap states that appear as an exponential continuous tail just below the conduction band, being associated to structural disorder and unbalanced Ti atoms on the surface.^50^ The PL spectrum of P25 shows a wide band, which can be deconvoluted into three different peaks centered at 442 nm (blue), 530 nm (green), and 600 nm (yellow-red), respectively. The blue peak is attributed to transitions between the shallow continuous states and the valence band, while the green and red PL peaks involve deep intermediate gap states originated by intrinsic point defects, such as oxygen vacancies, and are typical of anatase TiO_2_.^50,51^ In nanostructured TiO_2_, oxygen vacancies are located close to the surface, and their densities can be affected by environmental conditions. It has been widely reported that O_2_ adsorption results in a significant reduction of the PL intensity of anatase TiO_2_, and the opposite occurs upon O_2_ desorption.^19^

Sample B-0-TiO_2_-550 presents a PL spectra similar to that of P25, but with a more intense blue band, indicating a less efficient charge relaxation toward trapped states near the surface. On the other hand, B-5-TiO_2_-550 exhibits a drastic quenching of all PL bands (red line in Figure 6a). The absorbance spectra of these two samples (see Figure S7) are very similar, indicating that absorption is not affected by B incorporation (absorption in the visible region does not occur), ruling out one of the possible mechanisms by which B can modify the activity of TiO_2_ (substitution of oxygen atoms would produce the TiO_2_ band gap narrowing). However, the mentioned PL quenching reveals an overall reduction in the recombination rate of photo-excited electrons and holes, which can be explained by a more efficient charge separation and transfer toward the surfaces.^52−55^ This supports the interpretation of its highest photocatalytic activity. The suppression of PL allows the observation of several sharp features present in the blue region. These findings correspond to the resonant Raman spectrum of the sample and have been plotted in more detail in Figure 6b. The Raman spectrum is dominated by three very intense peaks (below 1000 cm^–1^) labeled as the principal vibrational modes of anatase TiO_2_. The low-frequency *E*_g_ mode of anatase is not detected as it has been cut off by the Raman edge filter. There are no traces of rutile modes, further confirming that anatase is the most abundant crystallographic phase in these materials. Similar peaks are observed in the spectrum of sample B-0-TiO_2_-550 but, surprisingly, not in the case of P25, which shows no resonant Raman signal. Finally, sample B-5-TiO_2_-550 exhibits two additional Raman bands at higher wavenumbers. These bands, which are not seen in the Raman spectrum performed with green laser excitation (not shown), have been linked to vibrational H–O–H bending modes at 1600 cm^–1^ and to the stretching vibrational modes of hydroxyl groups between 3000 and 3500 cm^–1^.^56^ These observations would indicate, on the one hand, the chemisorption of water molecules and, on the other hand, the production of hydroxyl radicals under UV irradiation.^57^ The presence of the latter band only in the Raman spectrum of sample B-5-TiO_2_-550 could be linked to its higher photocatalytic efficiency. Although the presence of boron had no measurable effect in band gap narrowing, the highest photocatalytic performance of this sample could be linked to its best photocurrent response ability, the additional production of hydroxyl radical groups, and the lowest recombination rate of electron/hole pairs.

### 3.3 Effect of the Crystallization Temperature

Sample B-5-TiO_2_-550, the most active in the B-*X*-TiO_2_-550 series, has been selected to study the effect of the crystallization temperature on the properties of these photocatalysts. Thus, after the sol–gel synthesis, B-5-TiO_2_ sample has been heat-treated at 350 and 450 °C to be compared with B-5-TiO_2_-550. XRD patterns of the three B-5-TiO_2_-*T* photocatalysts (Figure S8 in Supporting Information) show the characteristic peaks of anatase (see Section 3.1.1), and only for B-5-TiO_2_-550, an incipient peak of rutile can be observed. Inset of Figure S8 shows the enlarged 22–30° 2θ range, revealing changes in the crystallinity of those samples. As expected, the peaks become broader as the crystallization temperature decreases, which means that the sample is less crystalline. Table 3 summarizes the main properties of the B-5-TiO_2_-*T* series and the measured propene conversion. Data corresponding to P25 are also included.

**Table 3:** Physicochemical Properties and Propene Conversion for the B-5-TiO_2_-*T* Samples and P25

sample	crystalline TiO_2_ (%)^a^	amorphous TiO_2_ (%)^a^	average crystallite size (nm)^b^	*S*_BET_ (m^2^/g)^c^	B at. (%)^d^	propene conversion (%)^e^
B-5-TiO_2_-550	A (75)–R (2)	23	A (25) R (19)	33	2.8	74
B-5-TiO_2_-450	A (76)	24	A (18)	29	5.4	84
B-5-TiO_2_-350	A (70)	30	A (11)	104	3.6	58
P25	A (73)–R (14)	13	22	55		61

a Determined as indicated in the characterization by XRD.

b Calculated from Scherrer’s equation.

c Calculated from N_2_ adsorption data.

d Estimated from XPS analysis.

e Determined by eq 9 at 30 mL/min.

Regarding the calculated percentage of crystalline and amorphous phases and the average crystallite size for B-5-TiO_2_-*T* samples (calculated following the same procedure as for the B-*X*-TiO_2_-550 set of samples, data included in Table 3), the B-5-TiO_2_-350 catalyst presents the lowest crystallinity (30% of the amorphous phase) and smallest average crystal size (11 nm), whereas the B-5-TiO_2_-550 has the highest crystallinity, even with a small amount of the rutile phase (2%). In addition, these data agree with specific surface areas (*S*_BET_) obtained for these materials (Table 3). The material prepared at 350 °C presents the highest surface area, which is consistent with the largest proportion of the amorphous phase and the smallest crystal size. Samples B-5-TiO_2_-450 and B-5-TiO_2_-550 show similar surface area and crystallinity, although the effect of the temperature is reflected in the higher average crystal size of the sample treated at 550 °C.

B-5-TiO_2_-350 and B-5-TiO_2_-450 were also studied by XPS, and they show features similar to sample B-5-TiO_2_-550 (see Figure S9 and Table S2 and Table S3 in Supporting Information). However, the sample prepared at 450 °C shows the highest boron atomic percentage, 5.4% (Table 3), meaning that the crystallization temperature most probably has an effect on the distribution of boron atoms on the TiO_2_ surface. Finally, data in Table 3 show that the catalyst crystallization temperature influences propene conversion, being B-5-TiO_2_-450 the most active sample (84% propene conversion) and B-5-TiO_2_-350 the least active one.

The higher activity of sample B-5-TiO_2_-450 compared to B-5-TiO_2_-550 (both have similar surface area) can be explained considering that the first contains pure anatase, of lower average crystal size, and larger amount of surface interstitial boron atoms. The lower activity of sample B-5-TiO_2_-350 can be related to the lower anatase content.

The photocatalytic propene oxidation under visible light using B-5-TiO_2_-450, B-5-TiO_2_-550, and P25 samples has also been studied. A previous blank experiment shows that, as expected, propene photolysis does not occur, while the tested photocatalysts led to relatively low propene conversion (around 15%), see Figure S10. The activity measured with visible light is probably mainly related to the contribution of UV radiation in the used visible lamp (see spectra in Figure S1, Supporting Information), although it seems that the three tested photocatalysts have some features by which the relative activity order under visible and UV light do not agree (Figure S10). In any case, the different lamp irradiances should be taken into account: UV irradiance of the visible lamp, 0.049 mW cm^–2^, is much lower than that of the UV-lamp, 22.4 mW cm^-2^, and the activity is not linearly related with irradiance.^58^ Nevertheless, the poor response under visible light is in agreement with the lack of band gap modification of TiO_2_ upon B incorporation (Figure S7 compares results of B-5-TiO_2_-550 and B-0-TiO_2_-550) and supports discarding substitutional B doping.

In order to explain the highest photocatalytic activity of sample B-5-TiO_2_-450 under UV light, the series of B-5-TiO_2_-*T* samples has been characterized by means of PEC and PL measurements (like in the case of the B-*X*-TiO_2_-550 set of samples discussed above).

#### 3.3.1 PEC and PL Characterization of B-5-TiO_2_-*T* Samples

**Figure 7:**
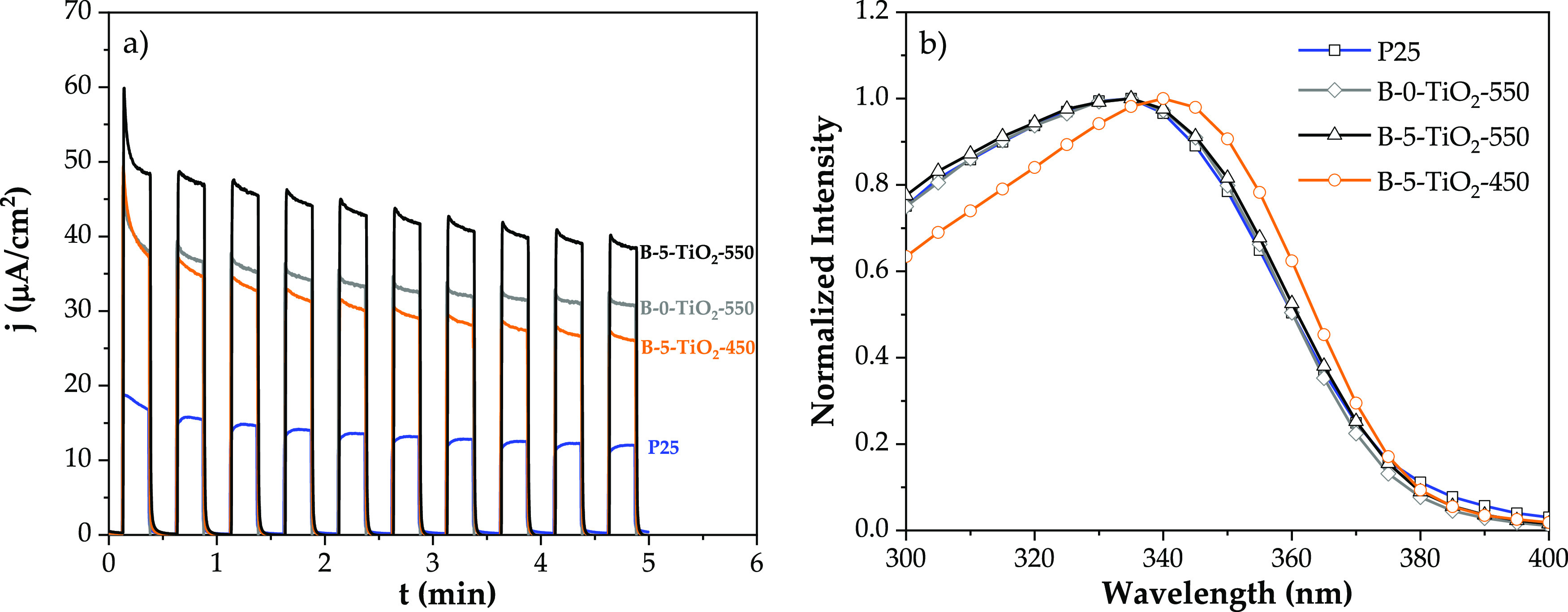
(a) Transient photocurrent measurements at a constant potential of 0 V vs Ag/AgCl and solar irradiation power of 70 mW cm^–2^ for electrodes containing P25, B-5-TiO_2_-450, B-0-TiO_2_-550, and B-5-TiO_2_-550. (b) Normalized photocurrent as a function of the irradiation wavelength at a constant potential of 0.4 V vs Ag/AgCl.

The PEC characteristics of the B-5-TiO_2_-450 electrode under CV conditions in the dark (not shown) are analogous to those of B-0-TiO_2_-550 and B-5-TiO_2_-550 (Figure 5). Under full solar light irradiation, all the specimens develop an induced photocurrent at positive potentials versus Ag/AgCl. The transient photocurrent response for those selected specimens is shown in Figure 7a. A high photocurrent could be related to an improved ability of this sample to decrease the radiative recombination of photogenerated electron/hole pairs. The intensity of light pulses shows an exponential decay, which relates to the capacitive filling of surface states. The dynamics shown by P25 is quite different from those of the sol–gel prepared photocatalysts in the considered time scale, again connecting with the link of higher surface area and charge accumulation. The B-5-TiO_2_-550 sample produces the highest intensity response. However, when the photocurrent is analyzed as a function of the light excitation wavelength using a monochromator (Figure 7b), samples B-0-TiO_2_-550 and B-5-TiO_2_-550, and P25 as well, show nearly identical wavelength-dependent profiles, while B-5-TiO_2_-450 has clearly a shift in its wavelength activity profile.

Furthermore, diffuse reflectivity (Figure S7) shows that regardless the crystallization temperature, all samples present a linear uprise of the absorption pointing to the same indirect band gap wavelength (427 nm), but B-5-TiO_2_-450 shows an Urbach tail (usually due to defects or loss of crystallinity) into the visible which is not present in the other two samples. This analysis reveals subtle differences between samples B-5-TiO_2_-450 and B-5-TiO_2_-550 and allows to propose that the wavelength shift of the photocurrent intensity maximum has a larger influence on the photocatalytic activity for propene oxidation under 365 nm UV light than the absolute intensity value.

**Figure 8:**
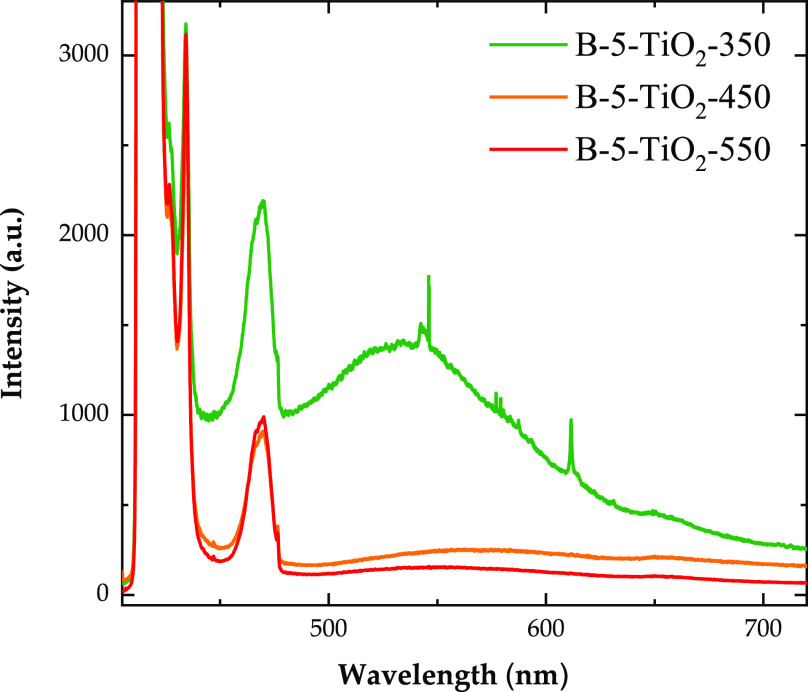
PL spectra of B-5-TiO_2_-550, B-5-TiO_2_-450, and B-5-TiO_2_-350 photocatalysts. The intensity of the spectra were normalized using the intensity of the anatase TiO_2_ Raman peaks, which should be proportional to the amount of excited material.

Finally, Figure 8 contains, for comparison purpose, PL spectra (excitation source of 405 nm) of B-5-TiO_2_-*T*.

Figure 8 reveals that the whole B-5-TiO_2_-*T* photocatalyst series exhibits a quenching of all PL bands (compared with the B-0-TiO_2_-550 sample shown in Figure 6). Thus, the reduction in the recombination rate of photo-excited electrons and holes when boron is (mostly interstitially) present is again confirmed. B-5-TiO_2_-450 and B-5-TiO_2_-550 samples show the lowest PL intensity within this series (lowest recombination rate of electron and holes), supporting the interpretation of their higher photocatalytic activities.

## 4 Conclusions

Nanostructured B-TiO_2_ photocatalysts (B-*X*-TiO_2_-550) with different amounts of boron (*X*) have been prepared by the sol–gel method and treated at different crystallization temperature (*T*). All the B-*X*-TiO_2_-550 catalysts have similar *S*_BET_ values (25–30 m^2^/g). The presence of boric acid during the TiO_2_ synthesis hampers the formation of rutile and, thus, the B-TiO_2_ samples mostly consist of anatase with minor quantities of rutile (<12%). XPS analysis reveals that B occupies interstitial positions (close to the surface), and when B is in excess, it is externally deposited as B_2_O_3_. The activity of the B-TiO_2_ samples for propene photo-oxidation is highly influenced by the boron content. Samples with low boron amount (*X* = 5 and 10) show the highest activity. The optimal behavior of these samples can be explained by the presence of interstitial B, together with a high crystallinity and high proportion of anatase. B_2_O_3_ deposits are formed in samples with higher boron loading, which cover the active titania surface with the subsequent activity decrease.

PEC and PL data reveal significant differences between samples. The highest photocatalytic performance achieved is related to the improvement of the photocurrent response ability (PEC), the production of hydroxyl radicals (Raman scattering), and the lower recombination rate of electron/hole pairs (PL), which points to a more efficient charge separation measurements.

The B-5-TiO_2_-450 sample is about 10% more active than B-5-TiO_2_-550. This improvement is related to the increased anatase content, lower average crystal size, and larger surface boron enrichment. Also, its intrinsic photo-activity suitably shifts to the wavelength of the light source (365 nm) and, as evidenced by PL data, it shows a low electron/hole recombination rate.

The present study highlights the importance of a controlled and accurate nanoscale doping of the titania surface in order to reach the suitable modification that improves the photocatalytic activity of TiO_2_. Also, it must be pointed out that the thorough characterization with complementary techniques has allowed to understand the effect of the interstitial B atoms in the photocatalytic behavior of the prepared samples.

## Supporting Information Available

Spectra of used lamps; TEM and EDX data of samples B-0-TiO_2_-550 and B-5-TiO_2_-550; HRTEM images of samples B-0-TiO_2_-550 and B-5-TiO_2_-550; XPS survey spectra of B-*X*-TiO_2_-550 samples; Ti 2p XPS spectra of B-*X*-TiO_2_-550 samples; PL spectra of B-*X*-TiO_2_-550; square root of absorbance data of samples B-5-TiO_2_-550, B-5-TiO_2_-450, and B-0-TiO_2_-550; XRD spectra of the B-5-TiO_2_-*T* samples; Ti 2p, O 1s, and B 1s XPS spectra of B-5-TiO_2_-*T* samples; propene conversion of B-5-TiO_2_-550 and B-5-TiO_2_-450 catalysts; textural properties of B-*X*-TiO_2_-550 samples and P25; binding energies of B 1s, O 1s, and Ti 2p in B-5-TiO_2_-*T* samples; and composition of samples B-5-TiO_2_-*T* determined by XPS (PDF)

## References

[ref1] Soni V.; Singh P.; Shree V.; Goel V. Effects of VOCs on Human Health; Springer, 2018; pp 119–142.

[ref2] Lillo-Ródenas M. A.; Bouazza N.; Berenguer-Murcia A.; Linares-Salinas J.; Soto P.; Linares-Solano A. Photocatalytic Oxidation of Propene at Low Concentration. Appl. Catal., B 2007, 71 (3-4), 298–309. 10.1016/j.apcatb.2006.10.004.

[ref3] Ouzzine M.; Lillo-Ródenas M. A.; Linares-Solano A. Photocatalytic Oxidation of Propene in Gas Phase at Low Concentration by Optimized TiO_2_ Nanoparticles. Appl. Catal., B 2013, 134-135, 333–343. 10.1016/j.apcatb.2013.01.035.

[ref4] Ghoshal A. K.; Manjare S. D. Selection of Appropriate Adsorption Technique for Recovery of VOCs: An Analysis. J. Loss Prev. Process Ind. 2002, 15, 413–421. 10.1016/S0950-4230(02)00042-6.

[ref5] Alberici R. M.; Jardim W. F. Photocatalytic Destruction of VOCS in the Gas-Phase Using Titanium Dioxide.Appl. Catal., B 1997, 14 (), 55–68. https://doi.org/10.1016/S0926-3373(97)00012-X.

[ref6] Yu H.; Zhang K.; Rossi C. Theoretical Study on Photocatalytic Oxidation of VOCs Using Nano-TiO_2_ Photocatalyst. J. Photochem. Photobiol., A 2007, 188, 65–73. 10.1016/j.jphotochem.2006.11.021.

[ref7] Nakata K.; Fujishima A. TiO_2_ Photocatalysis: Design and Applications. J. Photochem. Photobiol., C. 2012, 13, 169–189. 10.1016/j.jphotochemrev.2012.06.001.

[ref8] Fujishima A.; Zhang X. Titanium Dioxide Photocatalysis: Present Situation and Future Approaches. C. R. Chim. 2006, 9, 750–760. 10.1016/j.crci.2005.02.055.

[ref9] Jafari S.; et al. Biomedical Applications of TiO_2_ Nanostructures : Recent Advances. Int. J. Nanomed. 2020, 15, 3447–3470. 10.2147/IJN.S249441. PMC7234979 32523343

[ref10] Nyankson E.; Agyei-Tuffour B.; Asare J.; Annan E.; Rwenyagila E. R.; Konadu D. S.; Yaya A.; Dodoo-Arhin D. Nanostructured TiO_2_ and Their Energy Applications-a Review. ARPN J. Eng. Appl. Sci. 2013, 8, 871–886.

[ref11] Zhang L. Y.; Yang J. J.; You Y. H. Construction and Photocatalytic Performance of Fluorinated ZnO-TiO_2_ heterostructure Composites. RSC Adv. 2021, 11, 38654–38666. 10.1039/d1ra07757k. 35493257 PMC9044224

[ref12] Li Y.; Chen W.; Li L.; Ma M. Photoactivity of Titanium Dioxide/Carbon Felt Composites Prepared with the Assistance of Supercritical Carbon Dioxide: Effects of Calcination Temperature and Supercritical Conditions. Sci. China: Chem. 2011, 54, 497–505. 10.1007/s11426-010-4215-5.

[ref13] Ming L.; Youjing L.; Feitai C.; Xiao L.; Qiuju F. Electrically Enhanced Photocatalysis for Gas-Phase Benzaldehyde Degradation by Ordered Mesoporous Titania/Conductive Carbon Felts. Electrochim. Acta 2016, 216, 517–527. 10.1016/j.electacta.2016.08.054.

[ref14] Anpo M. The Design and Development of Highly Reactive Titanium Oxide Photocatalysts Operating under Visible Light Irradiation. J. Catal. 2003, 216, 505–516. 10.1016/S0021-9517(02)00104-5.

[ref15] Anucha C. B.; Altin I.; Bacaksiz E.; Stathopoulos V. N. Titanium Dioxide (TiO_2_)-Based Photocatalyst Materials Activity Enhancement for Contaminants of Emerging Concern (CECs) Degradation: In the Light of Modification Strategies. Chem. Eng. J. Adv. 2022, 10, 100262 10.1016/j.ceja.2022.100262.

[ref16] Ansón-Casaos M. T.; Sampaio A.; Jarauta-Córdoba A. M. T.; Martínez J. L.; Silva C. G.; Faria C.; Silva M. J. Evaluation of Sol–Gel TiO_2_ Photocatalysts Modified with Carbon or Boron Compounds and Crystallized in Nitrogen or Air Atmospheres. Chem. Eng. J. 2015, 277, 11–20. 10.1016/j.cej.2015.04.136.

[ref17] Devi L. G.; Kavitha R. A Review on Non Metal Ion Doped Titania for the Photocatalytic Degradation of Organic Pollutants under UV/Solar Light: Role of Photogenerated Charge Carrier Dynamics in Enhancing the Activity. Appl. Catal., B. 2013, 140–141, 559–587. 10.1016/j.apcatb.2013.04.035.

[ref18] Tan Y. N.; Wong C. L.; Mohamed A. R. An Overview on the Photocatalytic Activity of Nano-Doped- TiO_2_ in the Degradation of Organic Pollutants. ISRN Mater. Sci. 2011, 2011, 261219 10.5402/2011/261219.

[ref19] Jeong J. H.; Jung D. W.; Shin E. W.; Oh E. S. Boron-Doped TiO_2_ Anode Materials for High-Rate Lithium Ion Batteries. J. Alloys Compd. 2014, 604, 226–232. 10.1016/j.jallcom.2014.03.069.

[ref20] Xie K.; Zhang H.; Sun S.; Gao Y. Functions of Boric Acid in Fabricating TiO_2_ for Photocatalytic Degradation of Organic Contaminants and Hydrogen Evolution. Mol. Catal. 2019, 479, 110614 10.1016/j.mcat.2019.110614.

[ref21] Feng N.; Liu F.; Huang M.; Zheng A.; Wang Q.; Chen T.; Cao G.; Xu J.; Fan J.; Deng F. Unravelling the Efficient Photocatalytic Activity of Boron-Induced Ti3+ Species in the Surface Layer of TiO_2_. Sci. Rep. 2016, 6, 34765 10.1038/srep34765. 27708430 PMC5052528

[ref22] Zhang M.; Dai Y.; Zhang S.; Chen W. Highly Efficient Photocatalytic Activity of Boron-Doped TiO_2_ for Gas Phase Degradation of Benzene. Rare Met. 2011, 30, 243–248. 10.1007/s12598-011-0278-5.

[ref23] Grabowska E.; Zaleska A.; Sobczak J. W.; Gazda M.; Hupka J. Boron-Doped TiO_2_: Characteristics and Photoactivity under Visible Light. Procedia Chem. 2009, 1, 1553–1559. 10.1016/j.proche.2009.11.003.

[ref24] Li L.; Meng F.; Hu X.; Qiao L.; Sun C. Q.; Tian H.; Zheng W. TiO_2_ Band Restructuring by B and P Dopants. PLoS One 2016, 11, e0152726 10.1371/journal.pone.0152726. 27054763 PMC4824356

[ref25] Niu P.; Wu G.; Chen P.; Zheng H.; Cao Q.; Jiang H. Optimization of Boron Doped TiO_2_ as an Efficient Visible Light-Driven Photocatalyst for Organic Dye Degradation With High Reusability. Front. Chem. 2020, 8, 172 10.3389/fchem.2020.00172. 32232026 PMC7082229

[ref26] Wang X.; Wang K.; Wang H.; Wang Z.; Chen X.; Dai W.; Fu X. H_2_-Oxidation Driven by Its Behavior of Losing an Electron over B-Doped TiO_2_ under UV Irradiation. Phys. Chem. Chem. Phys. 2021, 23, 186–195. 10.1039/d0cp04039h. 33319875

[ref27] Dozzi M. V.; Artiglia L.; Granozzi G.; Ohtani B.; Selli E. Photocatalytic Activity vs Structural Features of Titanium Dioxide Materials Singly Doped or Codoped with Fluorine and Boron. J. Phys. Chem. C 2014, 118, 25579–25589. 10.1021/jp5084696.

[ref28] Zaleska A.; Grabowska E.; Sobczak J. W.; Gazda M.; Hupka J. Photocatalytic Activity of Boron-Modified TiO_2_ under Visible Light: The Effect of Boron Content, Calcination Temperature and TiO_2_ Matrix. Appl. Catal., B. 2009, 89, 469–475. 10.1016/j.apcatb.2009.01.005.

[ref29] Feng N.; Zheng A.; Wang Q.; Ren P.; Gao X.; Liu S. B.; Shen Z.; Chen T.; Deng F. Boron Environments in B-Doped and (B, N)-Codoped TiO_2_ Photocatalysts: A Combined Solid-State NMR and Theoretical Calculation Study. J. Phys. Chem. C 2011, 115, 2709–2719. 10.1021/jp108008a.

[ref30] Xu J.; Ao Y.; Chen M.; Fu D. Low-Temperature Preparation of Boron-Doped Titania by Hydrothermal Method and Its Photocatalytic Activity. J. Alloys Compd. 2009, 484, 73–79. 10.1016/j.jallcom.2009.04.156.

[ref31] Dozzi M. V.; Selli E. Doping TiO_2_ with P-Block Elements: Effects on Photocatalytic Activity. J. Photochem. Photobiol., C. 2013, 14, 13–28. 10.1016/j.jphotochemrev.2012.09.002.

[ref32] Boyjoo Y.; Sun H.; Liu J.; Pareek V. K.; Wang S. A Review on Photocatalysis for Air Treatment: From Catalyst Development to Reactor Design. Chem. Eng. J. 2017, 310, 537–559. 10.1016/j.cej.2016.06.090.

[ref33] Cazorla-Amorós D.; Alcañiz-Monge J.; De la Casa-Lillo M. A.; Linares-Solano A. CO_2_ as an Adsorptive to Characterize Carbon Molecular Sieves and Activated Carbons. Langmuir 1998, 14, 4589–4596. 10.1021/LA980198P.

[ref34] Rodriguez-Reinoso F.; Linares-Solano A. Microporous Structure of Activated Carbons as Revealed by Adsorption Methods; Thrower P. A., Ed.; Marcel Dekker Inc.: New York, NY, USA, 1989.

[ref35] Debye P.; Sherrer P. Interference of irregularity oriented particles in X ray. Phys. Z 1916, 17, 277–282.

[ref36] Zhang H.; Banfield J. F. Understanding Polymorphic Phase Transformation Behavior during Growth of Nanocrystalline Aggregates: Insights from TiO_2_. J. Phys. Chem. B 2000, 104, 3481–3487. 10.1021/jp000499j.

[ref37] Jensen H.; Joensen K. D.; Jørgensen J. E.; Pedersen J. S.; Søgaard E. G. Characterization of Nanosized Partly Crystalline Photocatalysts. J. Nanoparticle Res. 2004, 6, 519–526. 10.1007/s11051-004-1714-3.

[ref38] Greczynski G.; Hultman L. Compromising Science by Ignorant Instrument Calibration—Need to Revisit Half a Century of Published XPS Data. Angew. Chem., Int. Ed. 2020, 59, 5002–5006. 10.1002/anie.201916000. 31975485

[ref39] Ansón-Casaos A.; Hernández-Ferrer J.; Rubio-Muñoz C.; Santidrian A.; Martínez M. T.; Benito A. M.; Maser W. K. Electron Trap States and Photopotential of Nanocrystalline Titanium Dioxide Electrodes Filled with Single-Walled Carbon Nanotubes. ChemElectroChem 2017, 4, 2300–2307. 10.1002/celc.201700321.

[ref40] Cano-Casanova L.; Amorós-Pérez A.; Ouzzine M.; Lillo-Ródenas M. A.; Román-Martínez M. C. One Step Hydrothermal Synthesis of TiO_2_ with Variable HCl Concentration: Detailed Characterization and Photocatalytic Activity in Propene Oxidation. Appl. Catal., B. 2018, 220, 645–653. 10.1016/j.apcatb.2017.08.060.

[ref41] Cano-Casanova L.; Amorós-Pérez A.; Lillo-Ródenas M. Á.; Román-Martínez M. C. Effect of the Preparation Method (Sol-Gel or Hydrothermal) and Conditions on the TiO_2_ Properties and Activity for Propene Oxidation. Materials 2018, 11, 2227 10.3390/ma11112227. PMC6266794 30423926

[ref42] Aguilar T.; Navas J.; Alcántara R.; Fernández-Lorenzo C.; Gallardo J. J.; Blanco G.; Martín-Calleja J. A Route for the Synthesis of Cu-Doped TiO_2_ Nanoparticles with a Very Low Band Gap. Chem. Phys. Lett. 2013, 571, 49–53. 10.1016/j.cplett.2013.04.007.

[ref43] Shul’ga Y. M.; Matyushenko D. V.; Kabachkov E. N.; Kolesnikova A. M.; Kurkin E. N.; Domashnev I. A.; Brichkin S. B. Correlation between the E g (1) Oscillation Frequency and Half-Width of the (101) Peak in the X-Ray Diffraction Pattern of TiO_2_ Anatase Nanoparticles. Tech. Phys. 2010, 55, 141–143. 10.1134/S1063784210010238.

[ref44] Chen D.; Yang D.; Wang Q.; Jiang Z. Effects of Boron Doping on Photocatalytic Activity and Microstructure of Titanium Dioxide Nanoparticles. Ind. Eng. Chem. Res. 2006, 45, 4110–4116. 10.1021/ie0600902.

[ref45] Balcı S.; Sezgi N. A.; Eren E. Boron Oxide Production Kinetics Using Boric Acid as Raw Material. Ind. Eng. Chem. Res. 2012, 51, 11091–11096. 10.1021/ie300685x.

[ref46] Zhang W.; Yang B.; Chen J. Effects of Calcination Temperature on Preparation of Boron-Doped TiO_2_ by Sol-Gel Method. Int. J. Photoenergy 2012, 2012, 528637 10.1155/2012/528637.

[ref47] Stuart J.; Hohenadel A.; Li X.; Xiao H.; Parkey J.; Rhodes C. P.; Licht S. The Net Discharge Mechanism of the VB2/Air Battery. J. Electrochem. Soc. 2015, 162, A192–A197. 10.1149/2.0801501jes.

[ref48] Quesada-González M.; Boscher N. D.; Carmalt C. J.; Parkin I. P. Interstitial Boron-Doped TiO_2_ Thin Films: The Significant Effect of Boron on TiO_2_ Coatings Grown by Atmospheric Pressure Chemical Vapor Deposition. ACS Appl. Mater. Interfaces 2016, 8, 25024–25029. 10.1021/acsami.6b09560. 27622709

[ref49] Fabregat-Santiago F.; Mora-Seró I.; Garcia-Belmonte G.; Bisquert J. Cyclic Voltammetry Studies of Nanoporous Semiconductors. Capacitive and Reactive Properties of Nanocrystalline TiO_2_ Electrodes in Aqueous Electrolyte. J. Phys. Chem. B 2003, 107, 758–768. 10.1021/jp0265182.

[ref50] Pallotti D. K.; Passoni L.; Maddalena P.; Di Fonzo F.; Lettieri S. Photoluminescence Mechanisms in Anatase and Rutile TiO_2_. J. Phys. Chem. C 2017, 121, 9011–9021. 10.1021/acs.jpcc.7b00321.

[ref51] Liu B.; Zhao X.; Yu J.; Parkin I. P.; Fujishima A.; Nakata K. Intrinsic Intermediate Gap States of TiO_2_ Materials and Their Roles in Charge Carrier Kinetics. J. Photochem. Photobiol., C. 2019, 39, 1–57. 10.1016/j.jphotochemrev.2019.02.001.

[ref52] Yu C.; Yu J. C. A Simple Way to Prepare C-N-Codoped TiO_2_ Photocatalyst with Visible-Light Activity. Catal. Lett. 2009, 129, 462–470. 10.1007/s10562-008-9824-7.

[ref53] May-Lozano M.; López-Medina R.; Rojas-García E.; Hernández-Pérez I.; Martínez-Delgadillo S. A. Characterization of B-TiO_2_ Synthesized under Different Conditions of Hydrolysis. J. Adv. Oxid. Technol. 2016, 19, 326–337. 10.1515/jaots-2016-0217.

[ref54] Sarkar A.; Khan G. G. The Formation and Detection Techniques of Oxygen Vacancies in Titanium Oxide-Based Nanostructures. Nanoscale 2019, 11, 3414–3444. 10.1039/c8nr09666j. 30734804

[ref55] Ma S.; Reish M. E.; Zhang Z.; Harrison I.; Yates J. T. Anatase-Selective Photoluminescence Spectroscopy of P25 TiO_2_ Nanoparticles: Different Effects of Oxygen Adsorption on the Band Bending of Anatase. J. Phys. Chem. C 2017, 121, 1263–1271. 10.1021/acs.jpcc.6b11714.

[ref56] Singh J.; Soni R. K. Fabrication of Hydroxyl Group-Enriched Mixed-Phase TiO_2_ Nanoflowers Consisting of Nanoflakes for Efficient Photocatalytic Activity. J. Mater. Sci.: Mater. Electron. 2020, 31, 12546–12560. 10.1007/s10854-020-03805-w.

[ref57] Abdullah A. M.; Garcia-Pinilla M.; Pillai S. C.; O’Shea K. UV and Visible Light-Driven Production of Hydroxyl Radicals by Reduced Forms of N, F, and P Codoped Titanium Dioxide. Molecules 2019, 24, 2147 10.3390/molecules24112147. PMC6600679 31174409

[ref58] Mazierski P.; Nadolna J.; Lisowski W.; Winiarski M. J.; Gazda M.; Nischk M.; Klimczuk T.; Zaleska-Medynska A. Effect of Irradiation Intensity and Initial Pollutant Concentration on Gas Phase Photocatalytic Activity of TiO_2_ Nanotube Arrays. Catal. Today 2017, 284, 19–26. 10.1016/j.cattod.2016.09.004.

