# Supplementary material for: Surface-Enriched Boron-Doped TiO2 Nanoparticles as Photocatalysts for Propene Oxidation
Source: ACS Appl Nano Mater. 2022 Aug 24;5(9):12527–39. doi: 10.1021/acsanm.2c02217 (PMC9513816; doi:10.1021/acsanm.2c02217)
Supplement: Supplementary file 1 — an2c02217_si_001.pdf [file an2c02217_si_001.pdf]

SUPPLEMENTARY MATERIAL

Surface-Enriched Boron-Doped TiO_2 Nanoparticles as Photocatalysts for Propene Oxidation

L. Cano-Casanova[1*], A. Ansón-Casaos[2], J. Hernández-Ferrer[2], A. M. Benito[2], W. K. Maser[2], N. Garro[3], M. A. Lillo-Ródenas[1], M. C. Román-Martínez[1*]

[1]*Grupo Materiales Carbonosos y Medio Ambiente, Departamento de Química Inorgánica e Instituto Universitario de Materiales (IUMA). Facultad de Ciencias. Universidad de Alicante. Ap.99. E-03080 Alicante, Spain.*

[2]*Instituto de Carboquímica, ICB-CSIC, Miguel Luesma Castán 4, 50018 Zaragoza, Spain.*

[3]*Institut de Ciència dels Materials (ICMUV), Universitat de València, 46980 Paterna, València, Spain.*

**Corresponding authors. Tel.: 0034 965903975; e-mails: mcroman@ua.es and lcanocasnova@gmail.com*

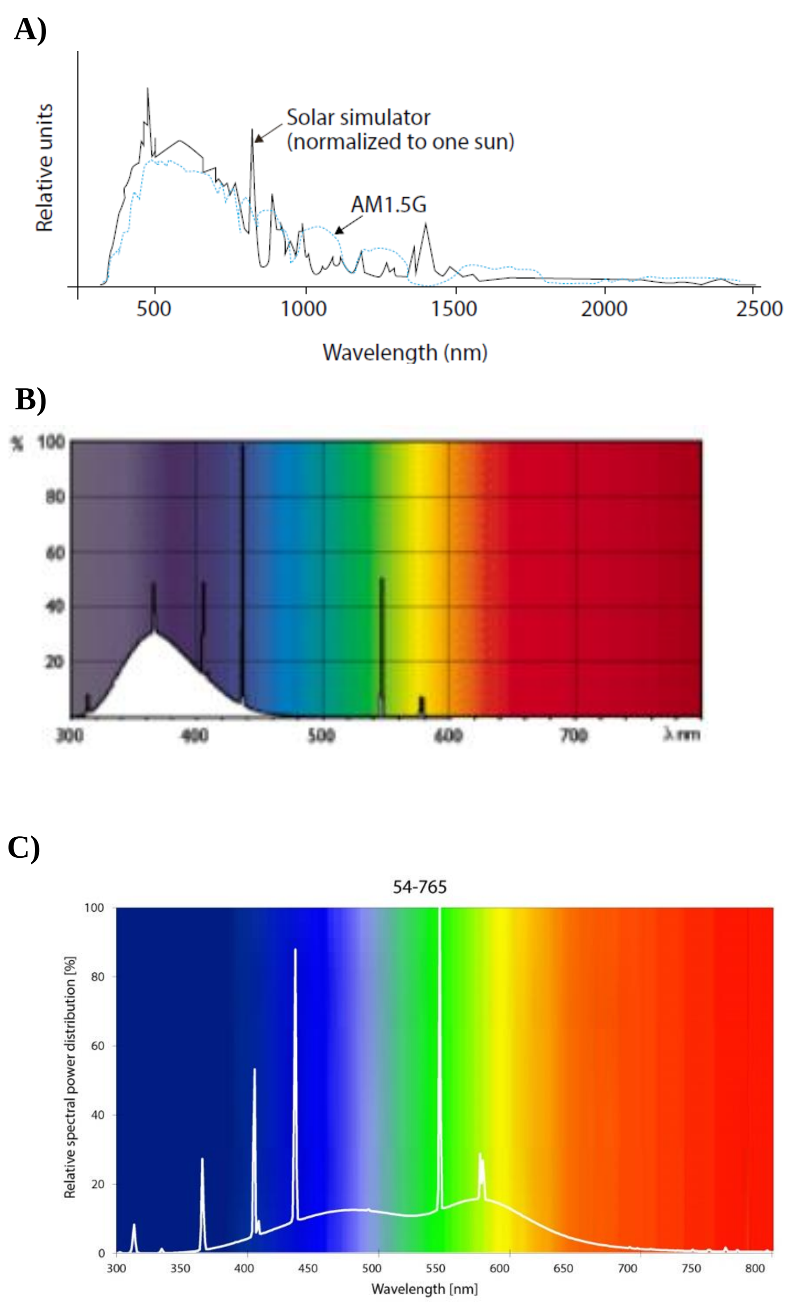

Figure S1. Spectra of lamps: A) solar simulator (AM1.5G), B)UV-A (TL8W/08 FAM), and C) visible (F8W/T5/54-765).

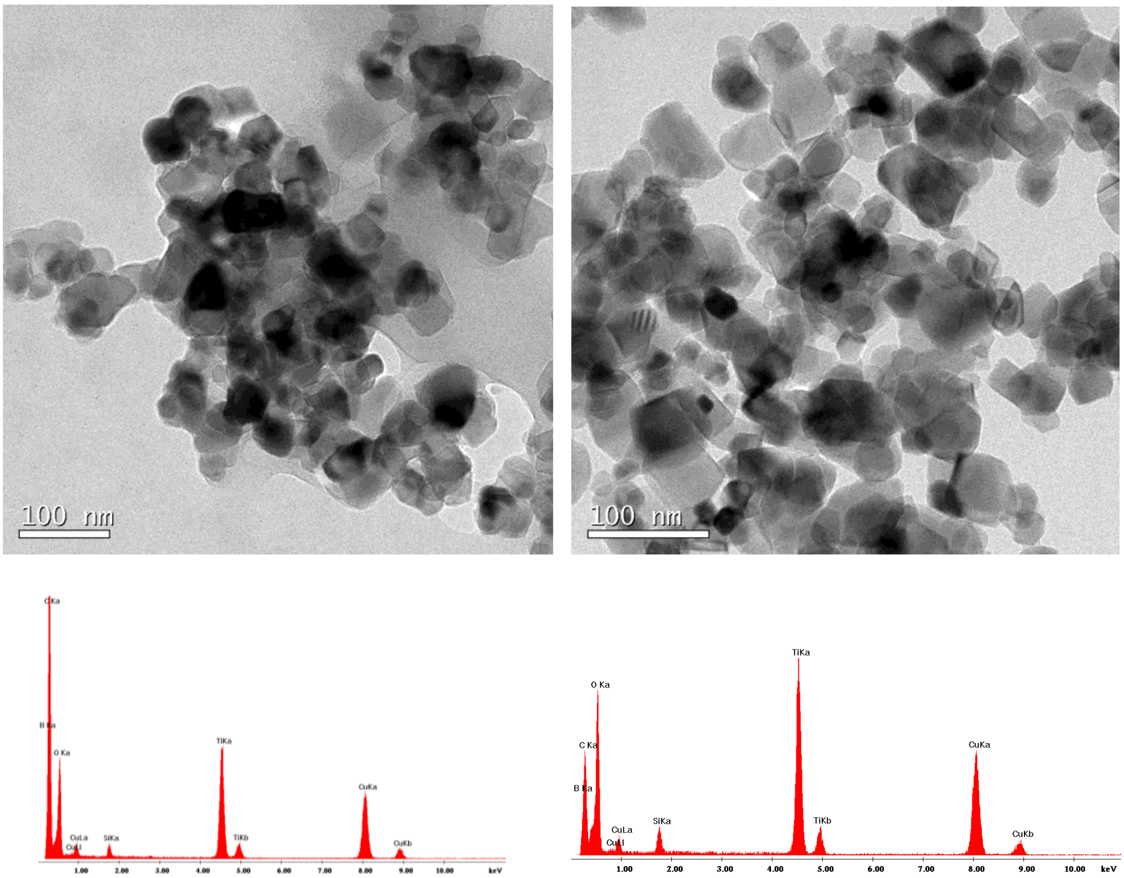

a) b)

Figure S2. TEM images and EDX data of samples: a) B-0-TiO_2-550 and b) B-5-TiO_2-550.

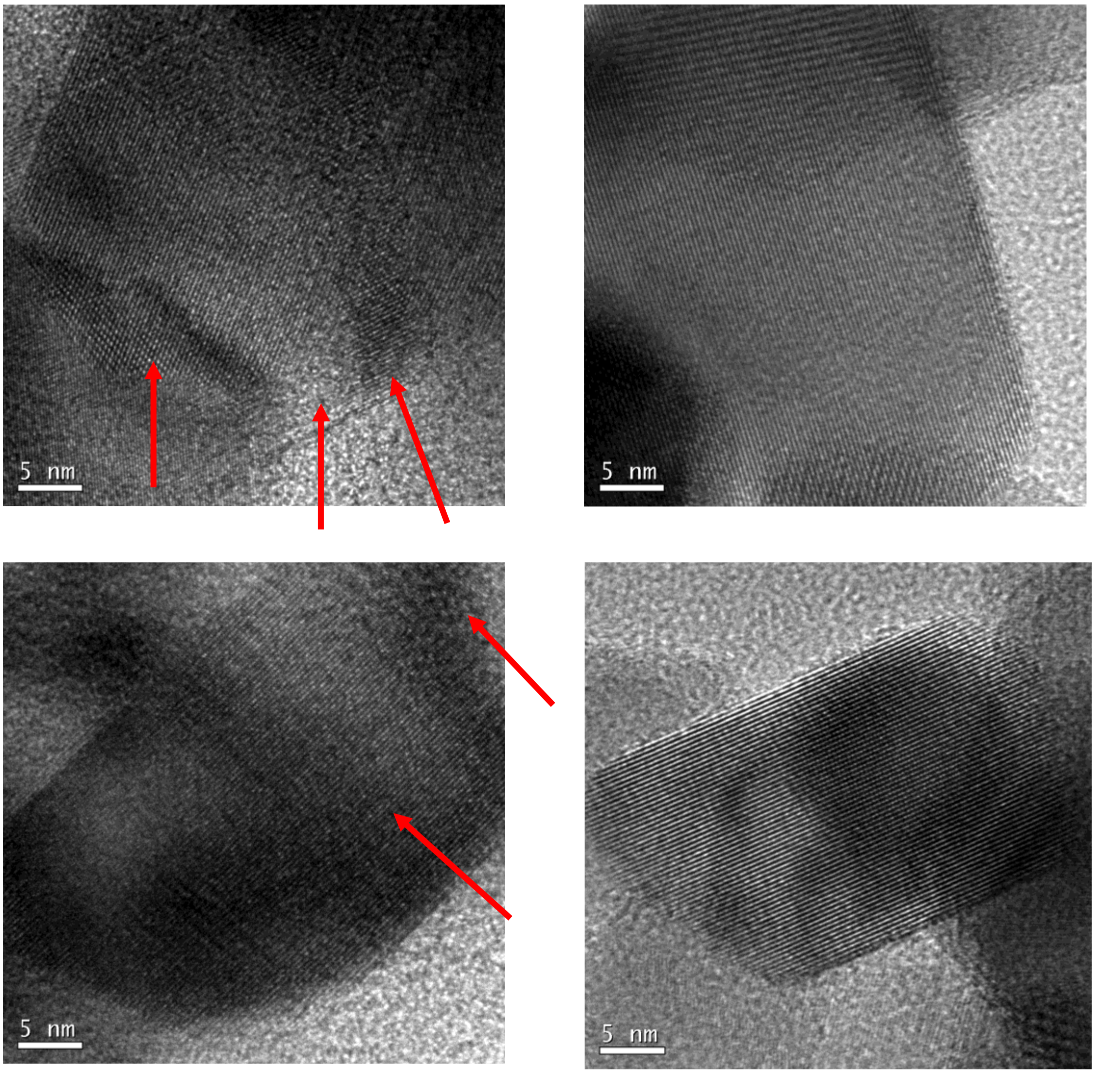

a) b)

Figure S3. HRTEM images of samples: a) B-0-TiO_2-550 and b) B-5-TiO_2-550. Red arrows point at different crystalline and amorphous domains observed in individual nanoparticles.

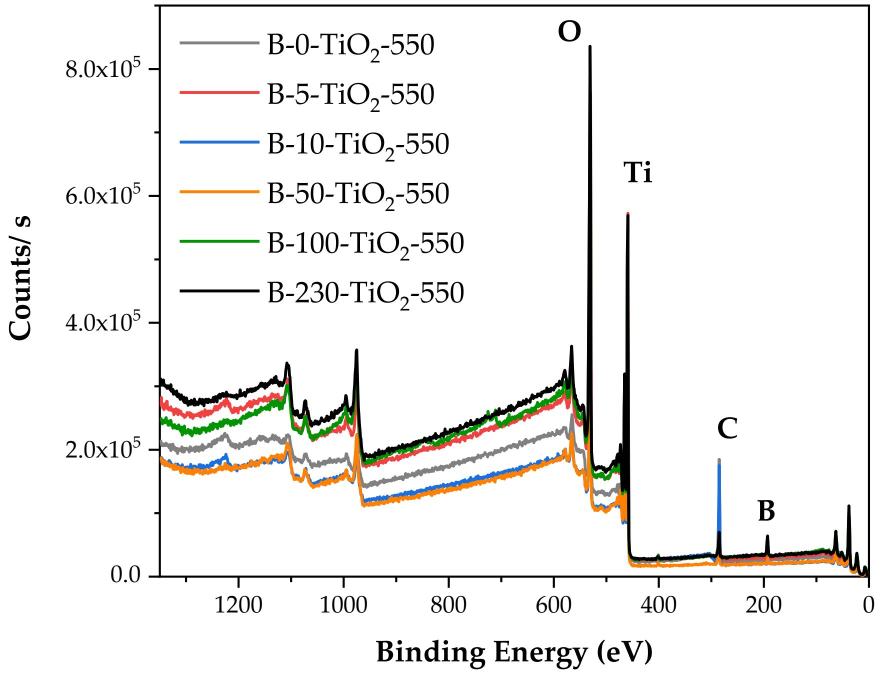

Figure S4. XPS survey spectra of B-X-TiO_2-550 samples.

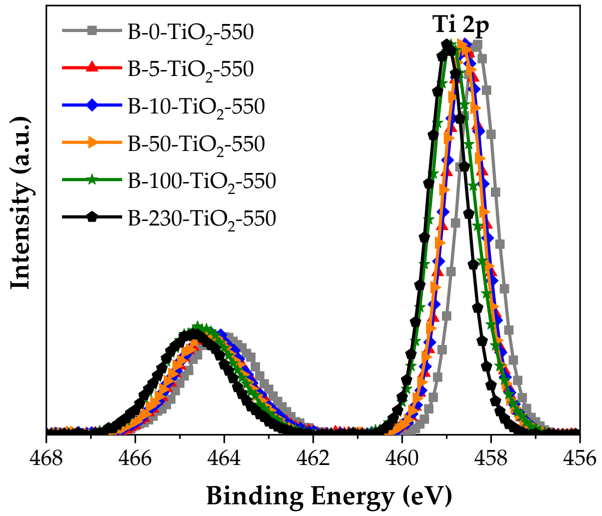

Figure S5. Ti2p XPS spectra of the B-X-TiO_2- 550 samples.

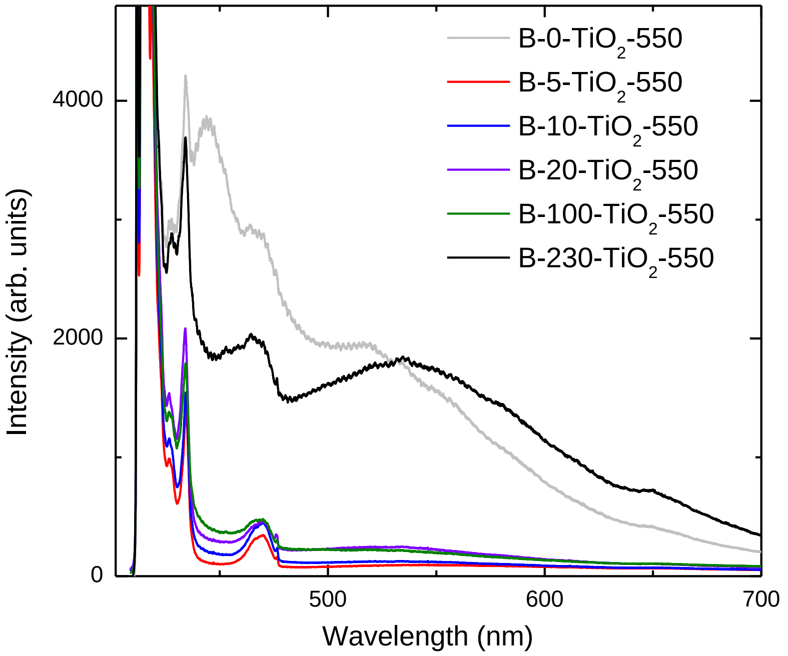

Figure S6. Photoluminescence spectra of the B-X-TiO_2-550 samples series. The intensity of the spectra has been normalized using the intensity of the anatase TiO_2 Raman peaks which should be proportional to the amount of excited material.

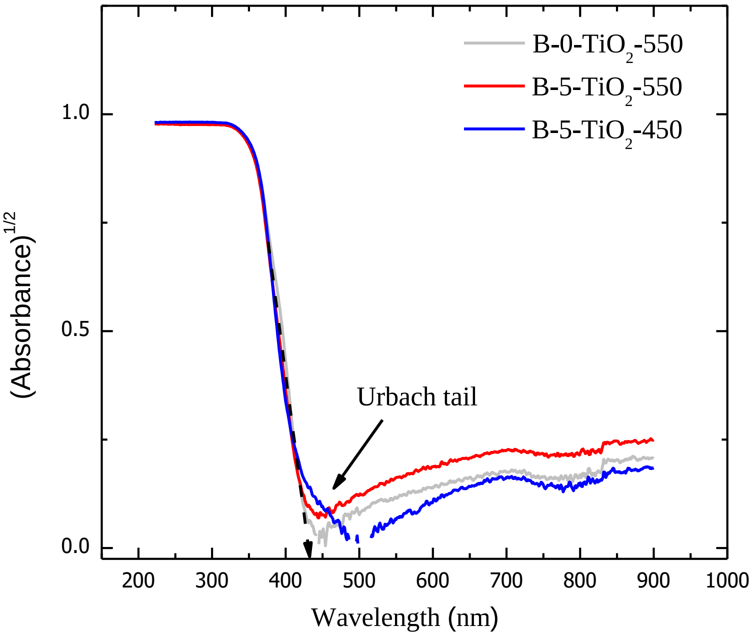

Figure S7. Square root of the absorbance of three catalysts with (B-5-TiO_2-550, B-5-TiO_2-450) and without (B-0-TiO_2-550) boron.

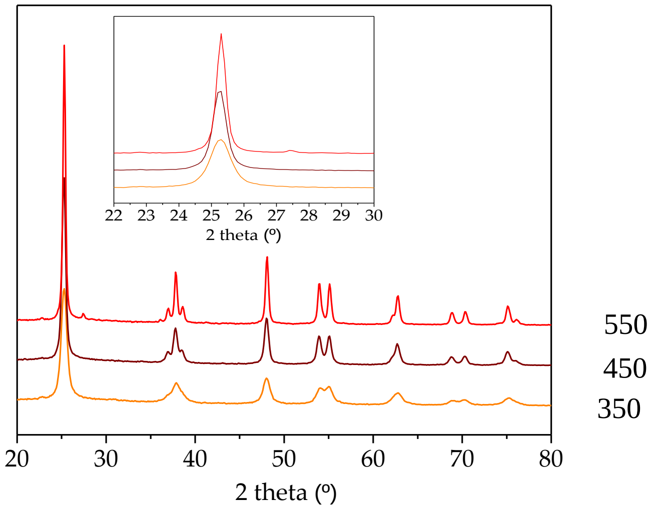

Figure S8. XRD spectra of B-5-TiO_2-T catalysts. Inset image: Amplification of the 24-30 º 2θ range.

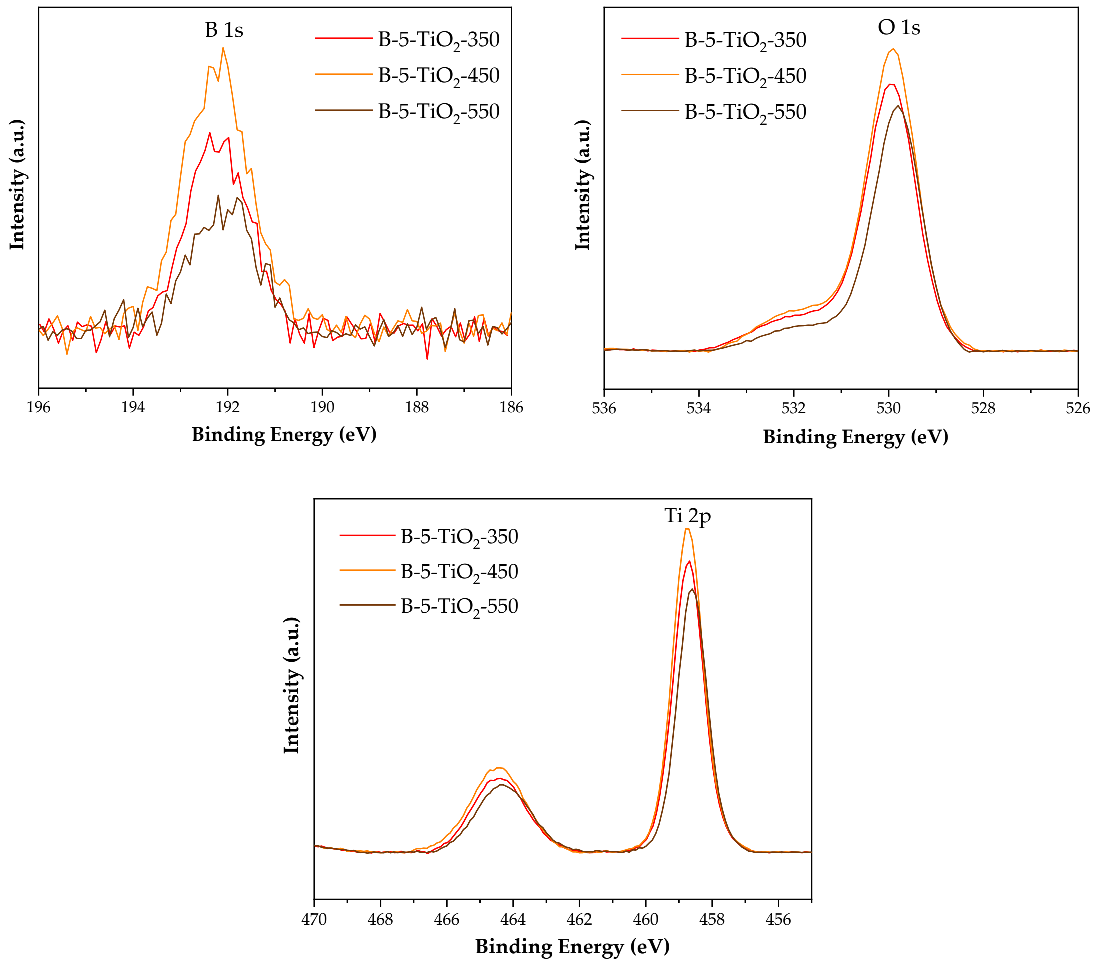

Figure S9. B 1s, O 1s and Ti 2p XPS spectra of the B-5-TiO_2-T samples series.

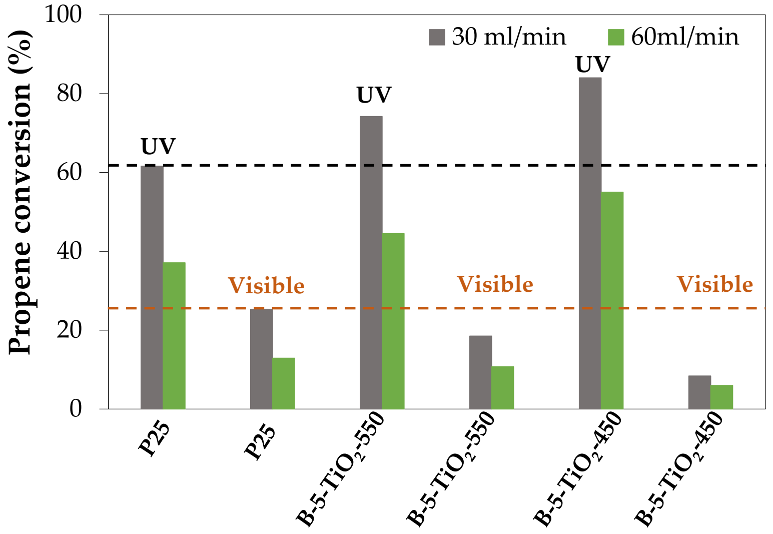

Figure S10. Propene conversion (%) at 30 and 60 ml/min for P25, B-5-TiO_2-550 and B-5-TiO_2-450 photocatalysts using UV and visible light.

Table S1. Textural properties for the B-X-TiO_2-550 series and P25.

Samples	S_{BET} (m^2/g)	V N_2 (cm^3/g)	V_{meso} (cm^3/g)
B-0-TiO_2-550	28	0.01	0.01
B-5-TiO_2-550	33	0.01	0.01
B-10-TiO_2-550	30	0.01	0.01
B-50-TiO_2-550	24	0.01	0.01
B-100-TiO_2-550	27	0.01	0.01
B-230-TiO_2-550	25	0.01	0.01
P25	55	0.02	0.07

Table S2. Binding energies of B1s, O1s and Ti 2p for B-5-TiO_2-T samples.

	Binding Energy (eV)					
Sample	B 1s	O 1s (I)	O 1s (II)	Ti $2p_{3/2}$	Ti $2p_{1/2}$	O 1s (I)/O 1s (II)
B-5-TiO_2-350	192.2	530.0	532.0	458.7	464.4	5.9
B-5-TiO_2-450	192.2	530.0	531.8	458.7	464.5	5.4
B-5-TiO_2-550	192.1	529.9	531.8	458.6	464.4	4.4

Table S3. Atomic percentages of B, Ti and O and atomic ratios derived from XPS for the B-5-TiO_2-T series. Nominal atomic percentage of B and ratio between XPS and nominal atomic B percentage.

Sample	B at%	Ti at%	O at%	B/Ti	O/Ti	B at% (nom)	B at %(XPS)/ B at % (nom)
B-5-TiO_2-350	3.6	18.8	53.4	0.2	2.8	0.14	25
B-5-TiO_2-450	5.4	22.3	62.6	0.2	2.8	0.14	38
B-5-TiO_2-550	2.8	20.1	55.0	0.1	2.7	0.14	20
